# OH-Detected Aromatic Microsolvation of an Organic NO Radical: Halogenation Controls the Solvation Side

**DOI:** 10.1021/acs.jpca.4c07744

**Published:** 2025-01-30

**Authors:** Elisabeth Sennert, Giovanni Bistoni, Martin A. Suhm

**Affiliations:** † Institute of Physical Chemistry, University of Göttingen, Tammannstrasse 6, 37077 Göttingen, Germany; ‡ Dipartmento di Chimica, Biologia e Biotecnologie, Università Degli Studi Di Perugia, Via Elce di sotto 8, 06123 Perugia, Italy

## Abstract

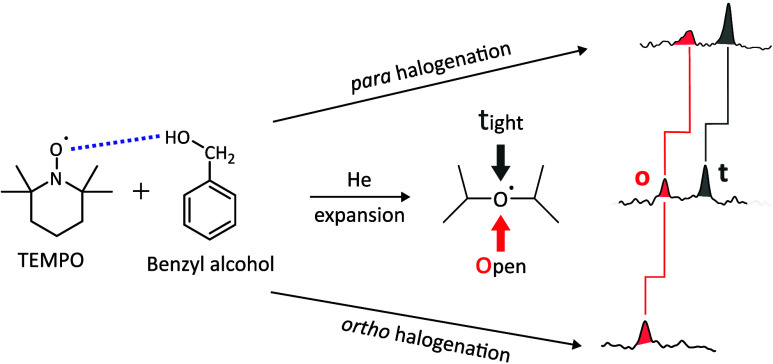

The persistent organic radical 2,2,6,6-tetramethylpiperidinyloxyl (TEMPO) protects its NO radical center by four methyl groups. Two of them are arranged tightly (t) on one side of the six-membered puckered heterocycle, and the other two more openly (o) on the other side. It is shown by OH stretching infrared spectroscopy in heated supersonic jet expansions that the hydrogen bond and aromatic ring of a first solvating benzyl alcohol have almost no preference for either side. An increased preference for the t side develops in *para*-halogenated benzyl alcohols, and it is inverted for *ortho*-halogenated benzyl alcohols. The experimental dependence on the actual halogen (Cl, Br, and I) is weak, whereas different quantum chemical approaches predict more or less pronounced trends along the halogen series. Some of the benzyl alcohol in the pre-expansion reservoir reduces the TEMPO radical to the closed-shell heterocyclic hydroxylamine TEMPO-H (1-hydroxy-2,2,6,6-tetramethylpiperidine), to the extent that the TEMPO-H···TEMPO complex is observed as an impurity.

## 1 Introduction

The NO group is a paradigmatic radical center, which has been studied intensely in its interaction with the environment, both in isolated form (nitric oxide) and embedded in an organic framework (aminoxyl). Nitric oxide is well suited for fundamental dynamic studies, such as gas phase energy transfer^1^ and reactivity^2^ or nonadiabaticity^3^ in its metal surface interaction,^4^ but also in the life sciences.^5^ The family of aminoxyl compounds helps in biological structure elucidation^6^ and provides valuable synthetic tools for redox reactions mediated by hydrogen transfer in the condensed phase,^7^ such as for copper- or enzyme-catalyzed benzylic alcohol oxidation.^8,9^ In both cases, the vibrational spectroscopy and dynamics of the NO group are sensitive to crucial details of the radical interaction with its binding partners.^3,10^ The low-frequency spectrum appears to be less sensitive to the solvation environment.^11^

**Figure 1:**
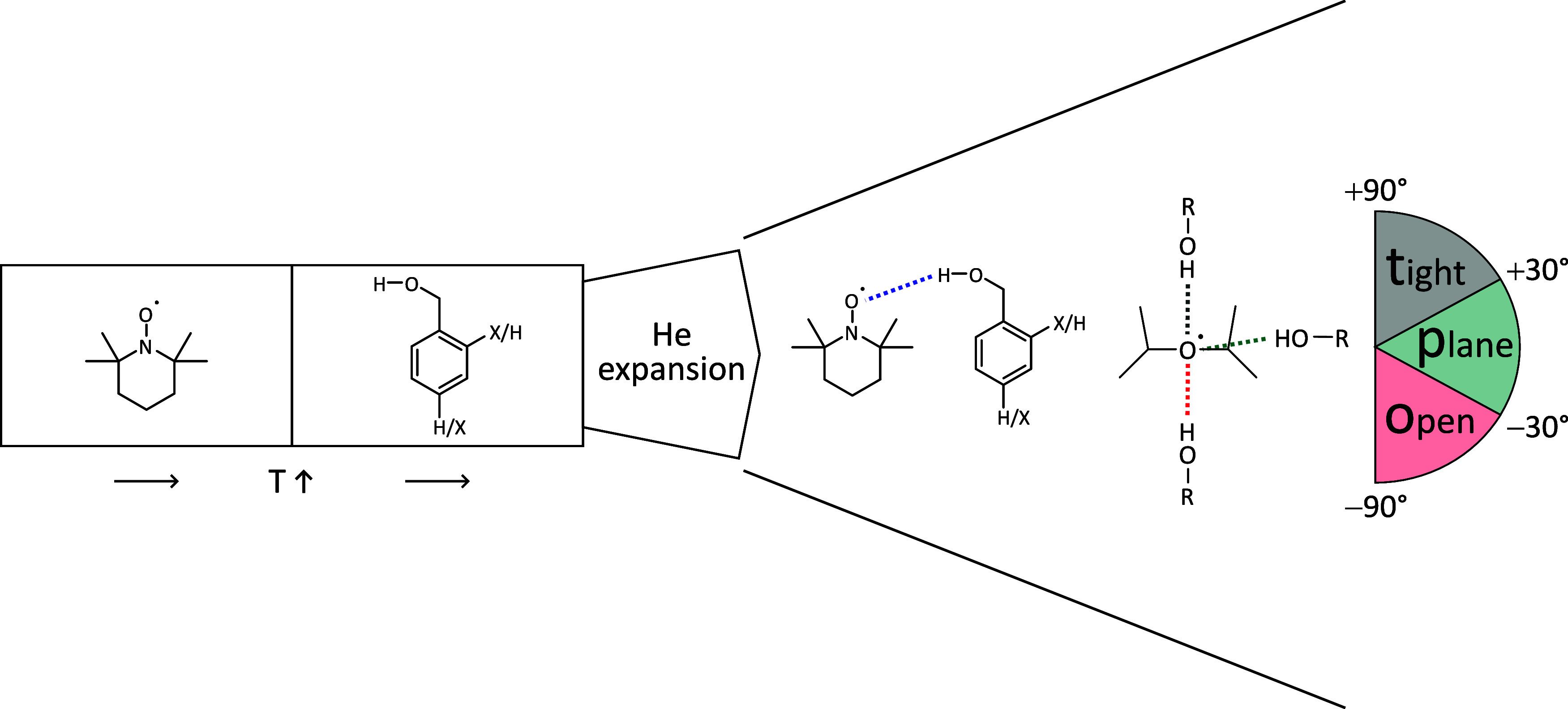
TEMPO and unsubstituted or *o*/*p*-halogenated benzyl alcohols are sequentially entrained into a helium flow by differential heating. Upon adiabatic expansion through a wedged nozzle into a vacuum, complexes form in which the OH group binds to the oxygen of the aminoxyl radical in up to three different sectors (t, o, p).

In the present work, we investigate the interaction of the 2,2,6,6-tetramethylpiperidinyloxyl (TEMPO) radical with a single aromatic solvent molecule via the vibrational spectrum of that solvent. We chose benzyl alcohol due to its interaction-sensitive IR chromophore (OH group) and its conformational flexibility, which allows for the simultaneous formation of a hydrogen bond to the radical center and a stacking interaction of the aromatic ring with the bulky piperidine unit, which is supported by London dispersion forces. Simpler protic solvents have been studied before^12,13^ and have revealed subtly modulated 3-fold protic solvation variants around the NO axis. The two faces of the α-methylated piperidine ring have been denoted as t for tight and o for open, describing the arrangement of the flanking methyl groups. For many protic solvents, there is also the option to solvate the NO bond in the piperidine plane (p; see Figure 1). While such interactions are relevant in bulk experiments, e.g., in EPR and NMR spectroscopy,^14−17^ it is appealing to study the isolated interactions in the gas phase. This allows for the most rigorous experimental tests of electronic structure methods and their ability to reproduce subtle structural and dynamic preferences in radical–solvent contacts. At temperatures where aminoxyl radicals have sufficient vapor pressure to be studied spectroscopically, such radical–solvent interactions are feeble and transient, whereas adiabatic cooling of the hot gas mixture can stabilize them and simplify their spectra. As TEMPO is one of the most volatile and elementary aminoxyl radicals, its investigation provides a good starting point.

By coexpanding the TEMPO radical with the aromatic alcohol seeded in a large excess of an atomic carrier gas through a heated slit nozzle, one can form 1:1 complexes of the two species. In the OH stretching range, they are easily distinguished from 0:2 complexes (alcohol homodimers), whereas TEMPO itself and its 2:0 homodimers only contribute to the neighboring CH stretching range. Single halogenation of benzyl alcohol in *para*-position does not break the solvent symmetry, whereas *ortho*-halogenation has been recently shown to lead to a weak internal hydrogen bond which is typically broken upon external hydrogen bonding.^18^ Ideally, one can thus expect a simple ternary (o, t, p) choice of any *ortho*-, *para*-, or nonhalogenated benzyl alcohol when docking on a TEMPO radical. Because the NO bond itself is fairly nondirectional in its solvation preference,^12^ the o:t:p abundance reflects the balance between steric hindrance near the hydrogen bond contact and dispersive attraction between the two ring moieties. However, the observed conformational preference is not entirely under thermodynamic control on the short time scale of a rarefied supersonic flow. Large barriers may hinder conformational interconversion^19^ such that one can define a conformational freezing temperature *T*_c_. *T*_c_ will be anywhere between the nozzle temperature (typically 340–410 K in the present experiments) and a temperature describing residual rotational excitation (typically 10–20 K for seeded slit jet expansions). Below *T*_c_, the interconversion is slow on the time scale of the supersonic jet expansion. Within these limitations, the jet experiments provide rather unambiguous evidence of the global minimum arrangement of a solvent molecule around the TEMPO radical. This conformational preference can be compared to theoretical predictions and thus help to rank quantum chemical methods such as density functionals but also highly correlated wave function methods in their ability to accurately predict the conformational landscape of TEMPO-solvent interactions. This is especially true if the theoretical prediction is qualitatively unaffected by zero point energy corrections.^20^

In the condensed phase and under suitable conditions, benzyl alcohol is particularly reactive with TEMPO.^21^ Under other conditions, some of the benzyl alcohol derivatives are even more reactive than the parent compound.^22^ If TEMPO reacts with the benzyl alcohol under the conditions of our experiments, a possible and spectroscopically detectable reaction product is TEMPO-H, which is the associated sterically hindered hydroxylamine. Its infrared spectrum has been characterized before^23^ and it may even form a complex with unreacted TEMPO which enables degenerate hydrogen transfer.^23^ Therefore, the analysis of the spectra presented in this work will also consider the possibility of impurities based on TEMPO-H and its complexes.

## 2 Methods

### 2.1 Experimental Techniques

A list of all used chemicals, their supplier, and purity can be found in the Supporting Information (SI, Table S5). For all experiments described in this work, the carrier gas helium is filled into a 69 L reservoir at a pressure of 1.5 bar and then pulsed through the heatable substance chamber where the investigated molecules are placed on a molecular sieve and can be picked up. The gas mixture is expanded through a heatable slit nozzle (60 mm × 0.2 mm) into the vacuum chamber, which is continuously pumped (500 m^3^ h^–1^) to ensure a low background pressure. The expansion is probed using a Bruker IFS 66v/s FTIR spectrometer, which includes CaF_2_ lenses, a KBr beamsplitter, and a ceramic glower as light source. For detection, an external L-N_2_-cooled Judson InSb detector is used. As a compromise between gas consumption and spectral width of the vibrational transitions, a spectral resolution of 2 cm^–1^ is chosen. The shown spectra are averaged over 200–540 scans (one scan per gas pulse). For more details regarding the setup, see refs (24,25).

Because two substances with low vapor pressures are combined in the present work, a second heatable substance chamber is added. The more volatile substance (here, always TEMPO) is added first (chamber P1) and the alcohol, second (chamber P2). The temperature increases from P1 over P2 to the heated nozzle tip (usually +20 K compared to P2). Zone temperatures for each measurement can be found in the SI (Table S6). Details for this modified setup can be found in ref (26) and especially in ref (27).

With increasing halogen size, an increasing temperature has to be set in the pre-expansion chamber to supply enough vapor pressure. This increases the likelihood of hydrogen transfer from the alcohol to TEMPO and thus the abundance of TEMPO-H.

### 2.2 Computational Techniques

If not described otherwise, all calculations in this work were carried out using ORCA 5.0.3.^28^ The CREST^29,30^ program was used to find possible conformers of the investigated systems which were then preoptimized using the B97–3c^31^ functional. All conformers up to a relative energy of 10 kJ mol^–1^ were reoptimized using the B3LYP^32−34^ functional with the def2-TZVP^35^ basis set, including D3 dispersion correction with Becke–Johnson damping (D3BJ)^36−40^ and three-body terms.^37^ After analyzing the resulting optimized structures according to structure type (o, t, p), only the most stable ones of each type in a window of about 2 kJ mol^–1^ were reoptimized and harmonically analyzed with the def2-QZVP basis set^35^ and are shown. This is justified by the simplicity of the spectra and the low interconversion barriers within a structure type (SI, Figures S1, S5, and S15). Only for the o structure type, multiple torsional isomers around the hydrogen bond were investigated in detail, because a strong dependence on the actual halogen was predicted. For iodine, an effective core potential (def2-ECP^41^) was used and an interesting aspect is to see whether there is a smooth transition between the Br (without) and I (with effective core potential) results in the comparison with experiment. Any discontinuity could be due to either enhanced relativistic effects in I or technical issues with the pseudopotential implementation. The transition state search was carried out by using the NEB-CI^42^ method at the B97-3c level to find possible transition state structures and further optimizing these structures at B97-3c and finally at the B3LYP/def2-TZVP level. For the UHF DLPNO–CCSD(T)^43,44^ calculations, including LED,^44,45^ the aug-cc-pVQZ^46−48^ basis set (aug-cc-pVQZ-PP^41^ for all iodine and partly for bromine calculations, see Section 3.6) and TightPNO settings were used. More details regarding the computational details and comments on the used isotope masses can be found in the Supporting Information (Table S5).

### 2.3 Nomenclature

The monomers studied in this work are abbreviated as B for benzyl alcohol, *p*X for benzyl alcohol with *para*-substituted halogen X (X = Cl, Br, I), and *o*X for the corresponding *ortho*-substitution. Homodimers are denoted by duplicating the abbreviation (e.g., *p*Cl*p*Cl). TEMPO is abbreviated T and the conformation of the 1:1 complex (BT or XT) with hydrogen bond between the alcoholic OH and the TEMPO oxygen atom around the NO bond is specified as o (open), t (tight), or p (plane), depending on whether the smallest positive CNO···H torsional angle is larger than 30° (o,t) or smaller (p), as illustrated in Figure 1. Primes (’, ”) are added to o, t, or p whenever energetically higher conformers with the same conformation type but slightly different orientation of the aromatic ring around the hydrogen bond are obtained for Cl (SI Table S3, Figures S17 and S18 for the *o*XT o). For the heavier halogens, this nomenclature is frozen such that o” for I is structurally similar to o” for Cl, even if it is now lower in energy. It is expected that higher energy conformations within a conformation type (o, t, or p) relax to the lowest energy choice in the adiabatic expansion. The full name of the 1:1 complex starts with the donor (B, *o*Cl, *p*Cl, *o*Br, *p*Br, *o*I, and *p*I), followed by the acceptor (T). The benzyl alcohol conformation is remarkably uniform for the systems studied in this work (gauche-oriented OH group, chiral) and because the radical is achiral, it does not have to be specified in the nomenclature. The 1:1 complexes illustrated in Figure 1 (right) would thus be sufficiently characterized as o/p/t variants of *o*XT/*p*XT.

## 3 Results and Discussion

Because the experimental infrared spectra display a limited complexity and the focus is on the ability of computational methods to predict the low-temperature gas phase experiments, we start with the presentation of the computational predictions for the 1:1 complexes with TEMPO before introducing and discussing the corresponding experimental spectra. We proceeded from plain benzyl alcohol over the more subtle *para*-halogenation to the more intrusive *ortho*-substitution.

### 3.1 TEMPO with Benzyl Alcohol

**Figure 2:**
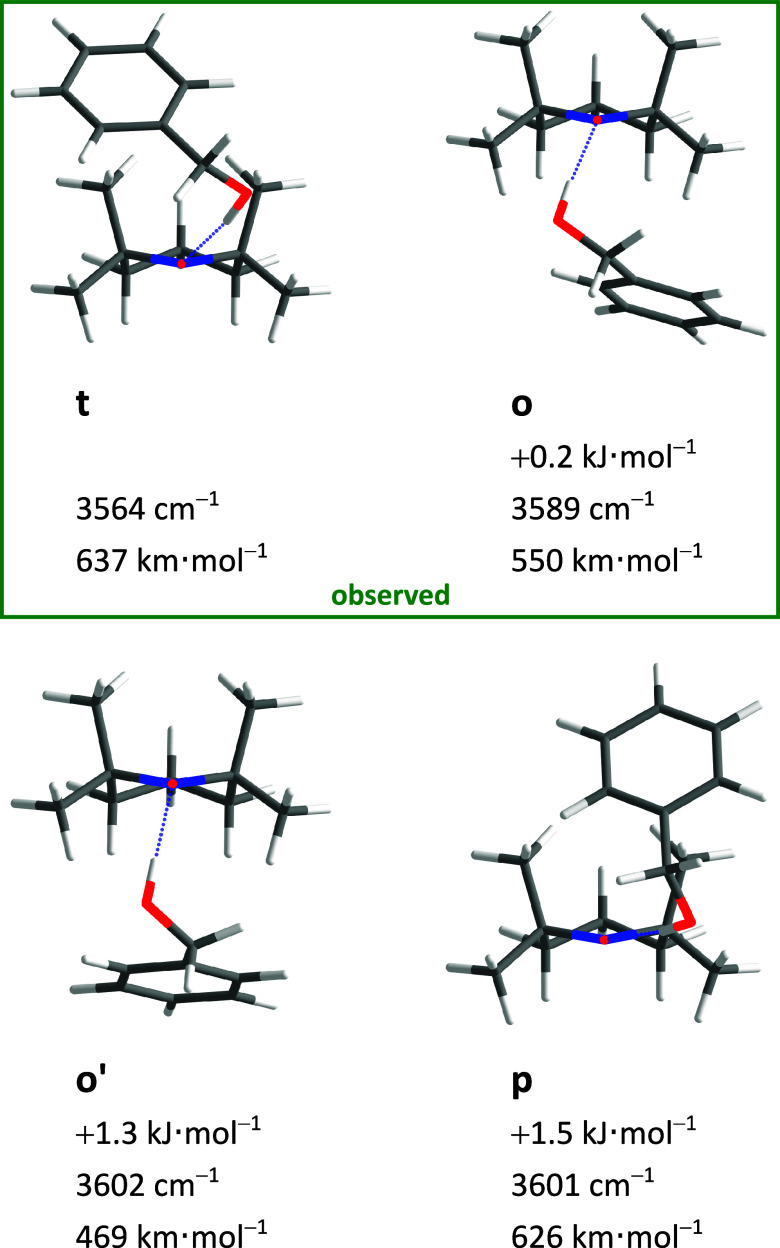
Optimized BT structures (*xyz* coordinates in SI Tables S8–S11) with harmonically zero-point-corrected relative energies, unscaled harmonic OH stretching wavenumbers, and harmonically approximated infrared intensities at the B3LYP-D3(BJ)/def2-QZVP level.

The conformational search for 1:1 complexes between benzyl alcohol and TEMPO revealed four low-lying conformations, displayed in Figure 2 together with their most relevant computed observables (relative zero-point level energy, harmonic OH fundamental stretching wavenumber, and harmonic infrared intensity). Their IR visibility is predicted to be similar, and they differ sufficiently in relative OH stretching wavenumber to distinguish the two higher energy conformers from the two lowest ones (t, o). Supported by the reaction profiles for conformational conversion (SI Figure S1), it is unlikely that the two higher ones (o’, p) survive the expansion such that the conformational search suggests the presence of two signals due to Boltzmann-populated conformers that are less completely interconverted. The relevant Boltzmann temperature is difficult to predict due to the nonequilibrium character of the supersonic expansion,^49^ but the computed interconversion barrier of less than 10 kJ mol^–1^ suggests that it falls below 200 K in a helium expansion. A more accurate assessment may have to include entropic differences between the two isomers, although rotational and low-frequency vibrational temperatures are expected to be low such that the zero-point energy difference will dominate the driving force for isomerization. The lower wavenumber signal (t) should be slightly stronger due to the associated subtle stability advantage and the somewhat higher predicted visibility.

**Figure 3:**
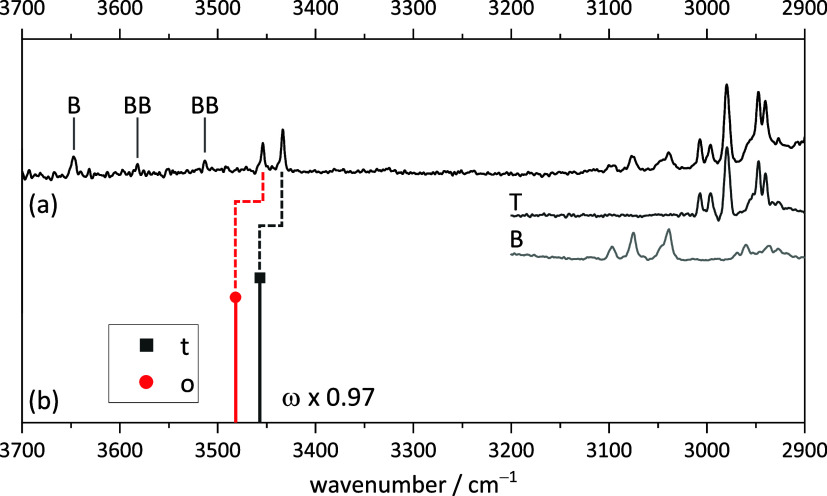
Measured Fourier transform infrared (FTIR) spectrum of (a) TEMPO with benzyl alcohol and (b) simulated stick spectra of the two most stable BT complexes (harmonic wavenumbers calculated at B3LYP-D3(BJ)/def2-QZVP level scaled by 0.97, relative intensity based on calculated IR band strength). B and BB mark the well-known^50^ benzyl alcohol monomer and homodimer signals. Additionally, the pure spectra of TEMPO and benzyl alcohol are shown in the CH region (3200–2900 cm^–1^). For experimental details, see SI Table S6.

This is precisely what is found in the experimental spectrum (Figure 3). The dominant peaks in the expected range (roughly predicted by scaling the harmonic wavenumber predictions to 0.97) reflect an (o/t) abundance ratio between 0.7 and 1.0. This is consistent with a sub-1 kJ mol^–1^ energy advantage for t and a conformational freezing temperature in the expected range of 100–200 K for a sub-10 kJ mol^–1^ interconversion barrier. All of the other features in the spectrum are easily explained. They are due to benzyl alcohol monomer (B) and dimers (BB)^50^ and the CH stretching range demonstrates the simultaneous presence of the radical (T) in comparison to single molecular component expansions. The absence of unexplained sharp OH spectral contributions in this and other spectra shown in the present work rules out major quantities of disolvate complexes, which might otherwise distort the relative abundance of the monosolvate complexes by selective aggregation. The expansions are sufficiently dilute, and the signal-to-noise ratio is still sufficient to reflect the coordination preference of the first alcohol to the radical.

This remarkable ability of scaled harmonic B3LYP-D3(BJ)/def2-QZVP predictions to correctly anticipate the unsymmetric spectral doublet could be coincidental. Already a 0.5 kJ mol^–1^ error in the relative energy would qualitatively remove the agreement. Therefore, it is imperative to secure this good agreement by chemical modification.

### 3.2 TEMPO with *para*-Chlorobenzyl Alcohol

**Figure 4:**
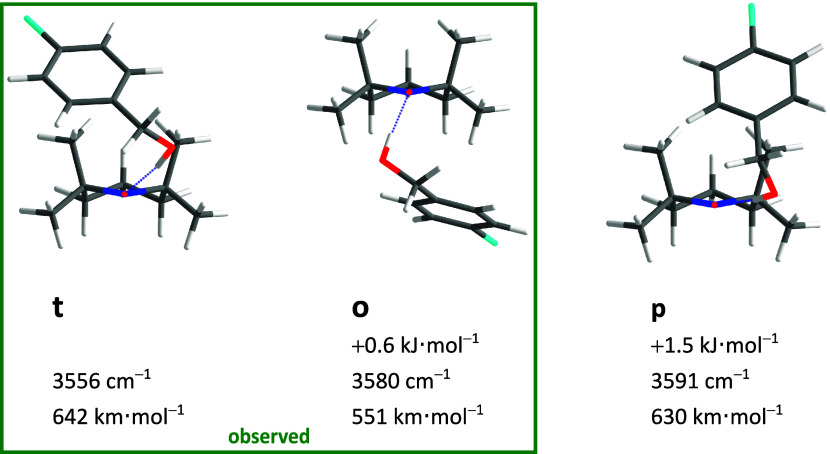
Optimized *p*ClT structures (*xyz* coordinates in SI Table S12–S14) with harmonically zero-point-corrected relative energies, unscaled harmonic OH stretching wavenumbers, and harmonically approximated infrared intensities at the B3LYP-D3(BJ)/def2-QZVP level. o’ is now outside the energy cutoff employed for the preoptimization and therefore not shown.

Among the most subtle chemical modifications conceivable for the BT complex is the *para*-chlorination of the aromatic ring. Figure 2 suggests that a Cl in the *para*-position will not be involved in the key intermolecular interactions. Therefore, the modulation of the complex structures and spectra is expected to be minor. Indeed, Figure 4 shows completely analogous t and o conformations of *p*ClT predicted at the same level of computation. The conformational shifts and intensity ratios are virtually the same; only the predicted energy difference has now increased from 0.2 to 0.6 kJ mol^–1^.

**Figure 5:**
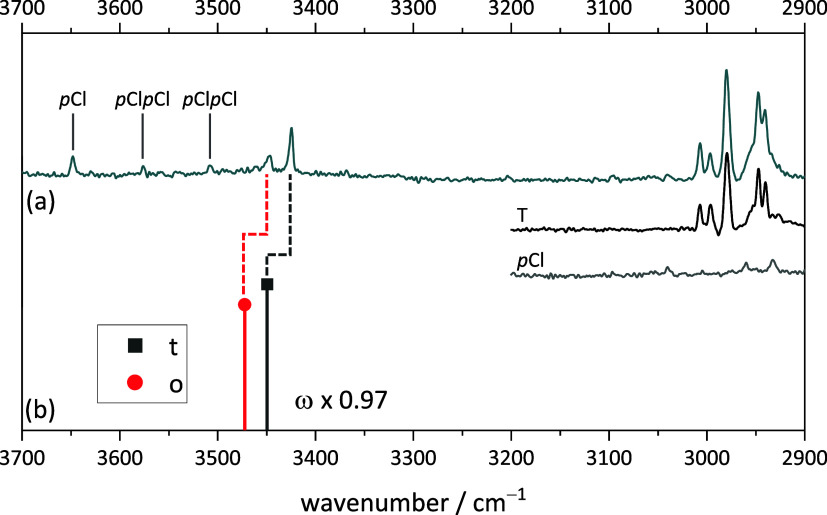
Measured FTIR spectrum of (a) TEMPO with *para*-chlorobenzyl alcohol and (b) simulated stick spectra of the two most stable *p*ClT complexes (harmonic wavenumbers calculated at the B3LYP-D3(BJ)/def2-QZVP level scaled by 0.97, relative intensity based on calculated IR band strength). *p*Cl and *p*Cl*p*Cl mark the well-known^51^*para*-chlorobenzyl alcohol monomer and homodimer signals. Additionally, the pure spectra of TEMPO and *para*-chlorobenzyl alcohol are shown in the CH region (3200–2900 cm^–1^). For experimental details, see SI Table S6.

While this is normally well within the expected accuracy of the harmonic density functional theory (DFT) approach, an increase in experimental intensity for the t conformation relative to BT would still provide valuable confirmation of the assignment and reduce the risk of fortuitous agreement. This expectation is supported by very similar interconversion barriers (SI Figure S5) and the absence of specific Cl interaction evidence in the NCI plots (SI Figures S3 and S4). Therefore, one can anticipate a spectral doublet with an accentuated intensity ratio, and this is precisely what is found in the experiment (Figure 5). Even the subtle spectral downshift of the doublet due to the influence of the Cl atom on the electron density of the aromatic ring (7–8 cm^–1^) is well predicted by theory (7–9 cm^–1^) such that the mismatch between theory and experiment stays almost constant.

### 3.3 TEMPO with *para*-Halogenated Benzyl Alcohols

Given the demonstrated ability of DFT calculations to predict the spectra of T radical complexes with benzyl alcohol before and after *para*-chlorination, it is of interest to study the Br- and I-homologues. The distance of the halogen from the main interaction center does not suggest major effects. However, the I atom requires the switch to a pseudopotential treatment, and the associated relativistic effects or approximations might introduce steps in the theoretical predictions which are not reflected in the experiment.

**Figure 6:**
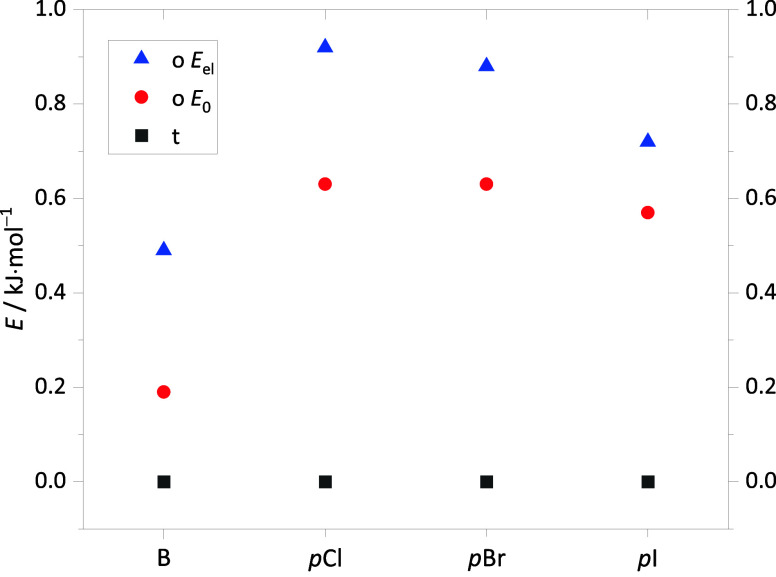
Computed relative energies of the main conformers for B, *p*Cl, *p*Br, and *p*I radical complexes, calculated at B3LYP-D3(BJ)/def2-QZVP level with (*E*_0_) and without (*E*_el_) ZPVE (zero-point vibrational energy).

**Figure 7:**
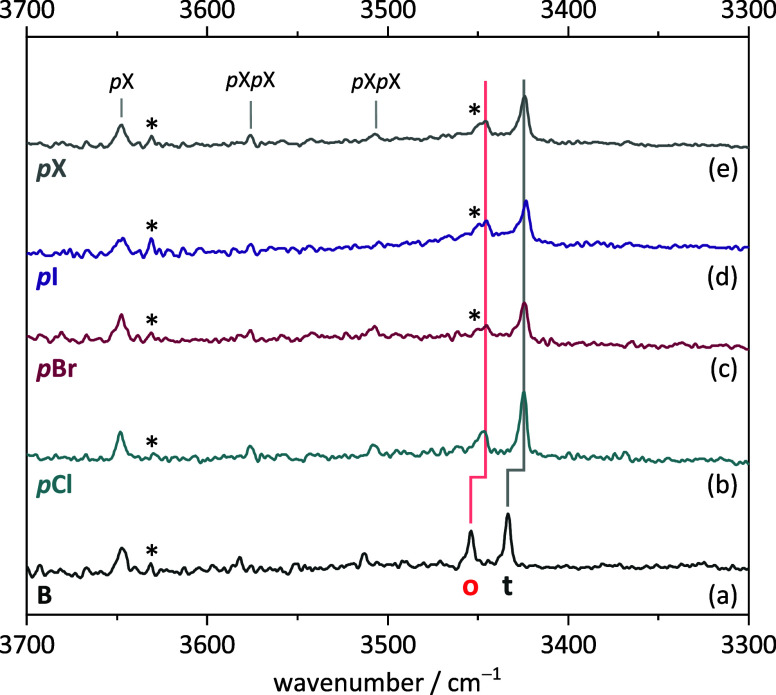
Measured FTIR spectra of TEMPO with (a) benzyl alcohol, (b) *para*-chlorobenzyl alcohol, (c) *para*-bromobenzyl alcohol, and (d) *para*-iodobenzyl alcohol. Trace (e) shows a scan weighted averaged spectrum out of (b–d) to illustrate the spectral similarities between the different halogens. The signals marked with an asterisk show TEMPO-H monomer and perhaps even TEMPO-H-TEMPO bands. For experimental details, see SI Table S6.

Figure 6 illustrates that the computed energetic effects across the halogen series are much more subtle than for the introduction of chlorine *para*-substitution in the first place. The destabilization of the o coordination largely persists, although it is slightly attenuated between Br and I. Similarly, the position of the t/o spectral doublet is predicted to remain similar across the halogen series, with a slightly increasing splitting (SI Figure S7). This again matches the experiment (Figure 7), although the experimental trends are even more subtle than the predicted ones. Intensity effects are more difficult to follow because of the growing TEMPO-H impurity (*) with increasing sample temperature, but qualitatively, they agree nicely with the expectation from the harmonic predictions.

The agreement is so close that an average over all spectra of *p*-halogenated benzyl alcohol coexpansions with T (uppermost trace in Figure 7) does not look significantly blurred. In summary, the *p*-halogenation of benzyl alcohol suggests that the performance of harmonic B3LYP predictions for the energetics and spectra of 1:1 benzyl alcohol complexes with TEMPO is systematic and reliable. All essential interactions appear to be captured in the correct proportions. A more critical test involves *ortho*-halogenation of the benzyl alcohol, where the halogen is closer to the radical center.

### 3.4 TEMPO with *ortho*-Chlorobenzyl Alcohol

The conformational landscape of *ortho*-chlorobenzyl alcohol itself is quite interesting and was the subject of a separate publication.^18^ As a monomer, it realizes a close balance between the intramolecularly hydrogen-bonded conformation and a nonbonded conformation where the heavy atoms fall in a plane, but in the homodimer, the hydrogen bond donor assumes a third CH_2_OH conformation which is similar to the one predicted here for the TEMPO complexes. It is a conformation in which the OH group points away from the chlorine atom. Therefore, it is likely that the alcohol conformation in the TEMPO complexes is not influenced by *ortho*-chlorination in a qualitative way.

**Figure 8:**
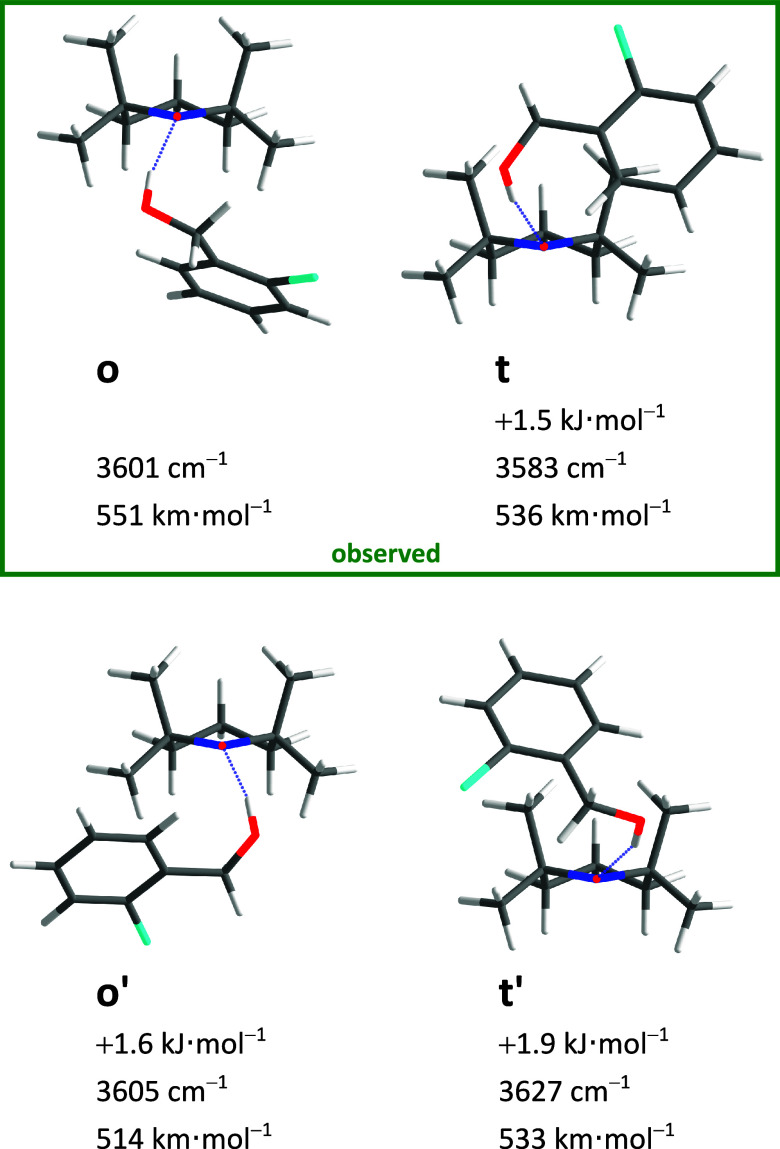
Optimized *o*ClT structures (*xyz* coordinates in SI Tables S15–S18) with harmonically zero-point-corrected relative energies, unscaled harmonic OH stretching wavenumbers, and harmonically approximated infrared intensities at the B3LYP-D3(BJ)/def2-QZVP level.

The harmonic DFT prediction for the coordination of TEMPO by *ortho*-chlorobenzyl alcohol confirms that and is summarized in Figure 8. The winner pair o,t is now followed closely by torsional variants of these two ring face options, o’ and t’. Coordination in the plane (p) is no longer an alternative in the 2 kJ mol^–1^ window.

The transition state search (SI Figure S15) suggests that relaxation from t’ to t should still be feasible, whereas relaxation from o’ to o may be somewhat hindered. Most importantly, o now falls energetically below t such that it represents the global minimum structure by a safe margin. Interconversion between t and o is facilitated by the *ortho*-chloro substituent, such that either one (o), two (o, t) or even three (o, t, o’) signals may be expected in the corresponding spectra. Spectral discrimination between o and t should be easy, whereas o and o’ are predicted to overlap.

**Figure 9:**
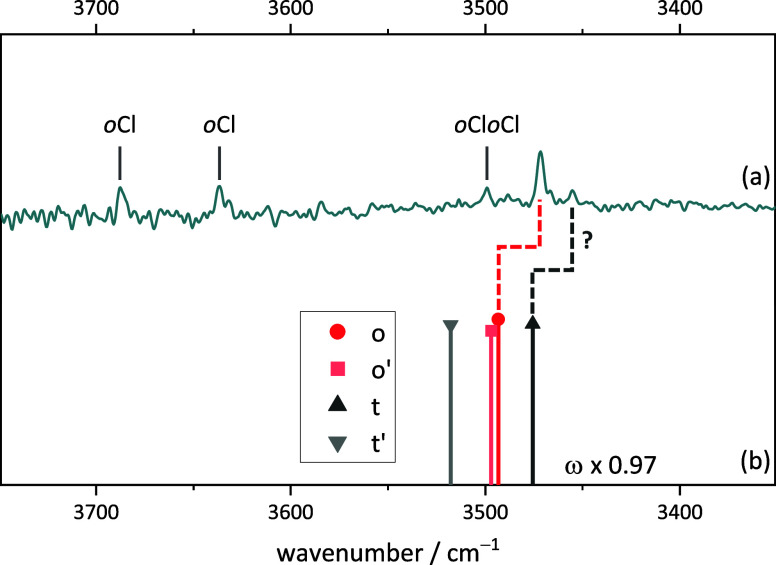
Measured FTIR spectrum of (a) TEMPO with *ortho*-chlorobenzyl alcohol and (b) simulated stick spectra of the four most stable *o*ClT complexes (harmonic wavenumbers calculated at the B3LYP-D3(BJ)/def2-QZVP level scaled by 0.97, relative intensity based on calculated IR band strength). *o*Cl and *o*Cl*o*Cl mark the *ortho*-chlorobenzyl alcohol monomer and homodimer signals.^18^ For experimental details, see SI Table S6.

The robust prediction is that a dominant o signal should be observed, somewhat downshifted compared to the 0.97-scaled prediction as in the previous cases. Any small t contribution should be observed further downshifted. This is what the experimental spectrum in Figure 9 shows. Whether the satellite band to lower wavenumber (?) is due to the t conformation is not entirely clear, but it would fit the prediction. Also, it has to remain open whether the satellite band to higher wavenumber is exclusively due to the alcohol homodimer^18^ or contains a small contribution from t’. The important result is that o is the preferred coordination face in the radical monosolvate complex, and one can now investigate whether this remains the case for heavier halogen atoms.

### 3.5 TEMPO with *ortho*-Halogenated Benzyl Alcohols

**Figure 10:**
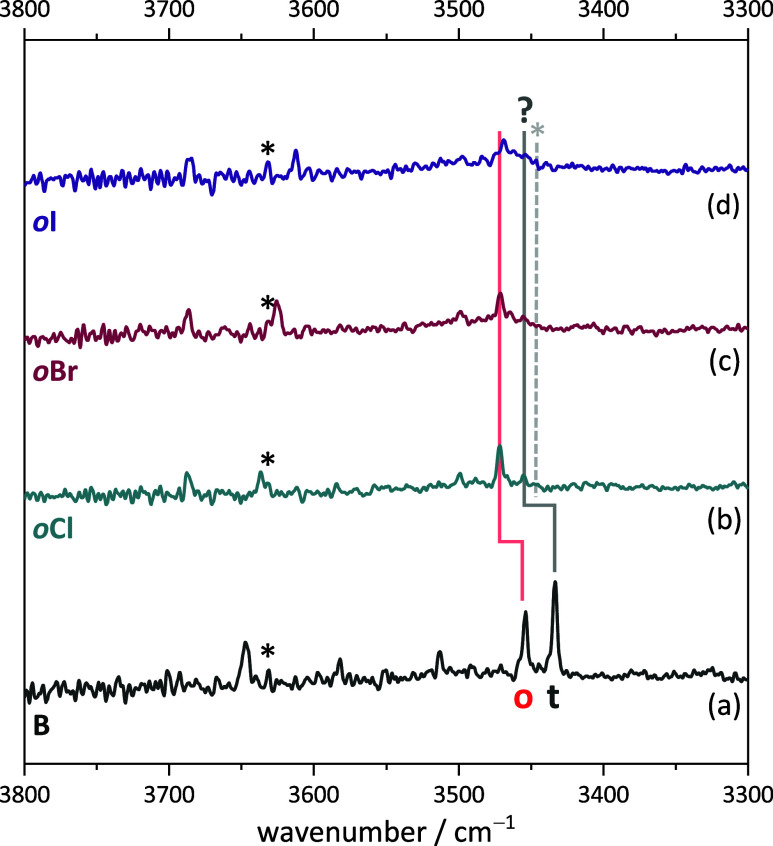
Measured FTIR spectra of TEMPO with (a) benzyl alcohol, (b) *ortho*-chlorobenzyl alcohol, (c) *ortho*-bromobenzyl alcohol, and (d) *ortho*-iodobenzyl alcohol. The t conformation is unquestionable in only (a). The * (asterisk) symbols mark TEMPO-H monomer traces (black) and the expected position of the dimer between TEMPO and the TEMPO-H impurity (gray). For experimental details, see SI Table S6.

Figure 10 shows the infrared spectra for the halogenation trend. As discussed elsewhere,^18^ one of the two alcohol monomer conformations is spectrally sensitive to the halogen, whereas the dominant homodimer is not. Therefore, we can focus on the spectral trend for the o conformation of the 1:1 complex, which is marked by a red vertical line.

There is a marked upshift from BT to the chlorinated variant *o*ClT which accompanies the stabilization of this conformation, but the position remains the same for *o*BrT and for *o*IT, the upshift is slightly attenuated. At the same time, the total signal intensity is reduced with increasing halogen size, which may be explained by the reduced volatility and increased decomposition tendency at the required elevated sample temperature.

**Figure 11:**
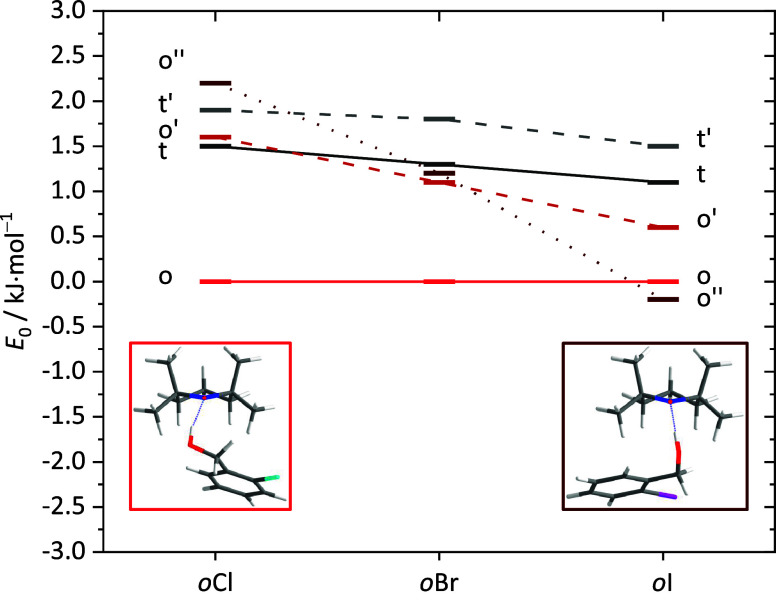
Energetic order of the *o*XT complexes at the B3LYP-D3(BJ)/def2-QZVP level with ZPVE. Any o coordination of the radical by the solvent is plotted in red colors, any t coordination in gray tones. The energies are shown relative to the o-coordination which wins for Cl.

Because the predicted relaxation pattern is less hierarchical than for the *para*-halogenated mixed complexes (SI Figure S5), any explanation of the experimental trend of the band position (upshift with chlorination, constant with bromination, and slight downshift to iodination) has to include several options. As Figure 11 shows, a fifth conformational option (o″) needs to be added into the analysis beyond the four shown in Figure 8 for Cl, because it gains in energetic competitiveness for the higher halogens. For I, it even becomes the predicted global minimum structure, whereas it is the least stable of the five conformations for Cl. The two competing dimer conformations o and o″ are shown in the bottom part of Figure 11. They can be easily distinguished by a range of alcohol-ON torsional angles (SI Table S3). o is characterized by nearly orthogonal NO and CC bonds, where CC is the aromatic bond connecting the two aromatic substituents. In o″, these two bonds are nearly parallel. As a consequence, o″ allows the halogen to interact more directly with the piperidine ring than o. It appears plausible that a heavier halogen maximizes this intermolecular interaction, but the monotonic experimental spectra do not support such a switch from o to o″ when iodine is involved.

**Figure 12:**
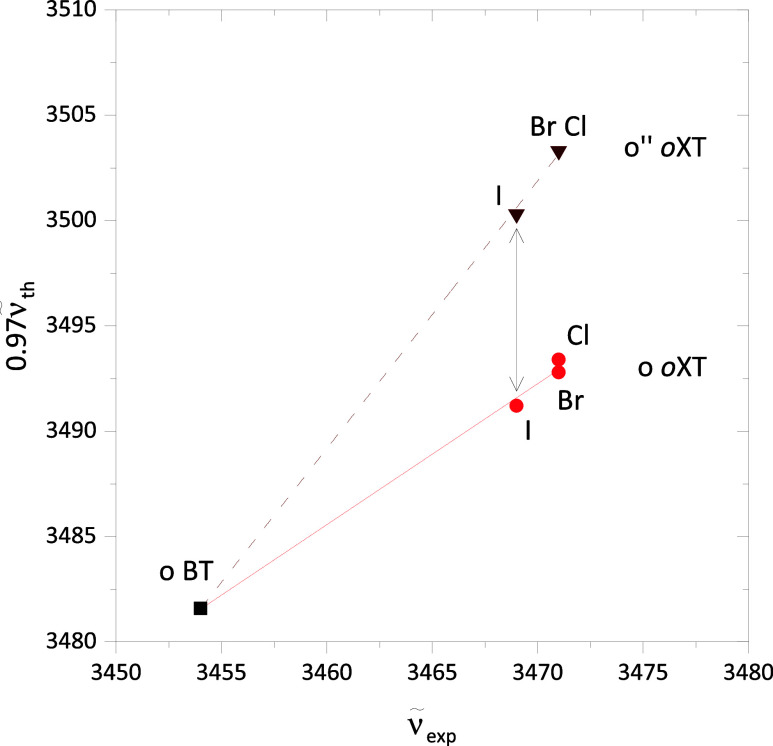
Theoretically predicted OH stretching wavenumber 0.97ν̃_th_ as a function of experimental OH stretching wavenumber ν̃_exp_ for different assignment scenarios of complexes where the alcohol is on the o(pen) side of the radical. The solid line connects a uniform o assignment (circles), and the dashed line a uniform o″ assignment (inverted triangles) for the halogens to the o BT complex transition. A switch from o to o″ for I (double arrow) is not in line with experiment.

If the predicted subtle switch from o to o” were real, it would likely manifest itself in a spectral shift >10 cm^–1^ (SI Figure S19), because the hydrogen bond is sensitive to such details. Figure 12 correlates the experimentally observed wavenumbers for the open conformers with the theoretically predicted (scaled) ones. One can see that a consistent o interpretation across all four species matches theory well (solid connecting line), although the shift from B to *o*X is larger in experiment than in theory (slope < 1). A consistent o” interpretation for all halogenated complexes is also reasonable (dashed connecting line, slope > 1). However, a switch from o to o” between Br and I (double arrow) is not supported by the experimental trend. This discrepancy between conformational homogeneity in experiment and conformational preference switch in the DFT prediction will be investigated in the next section at a higher computational level for the electronic energy.

### 3.6 CCSD(T) and LED Results

**Figure 13:**
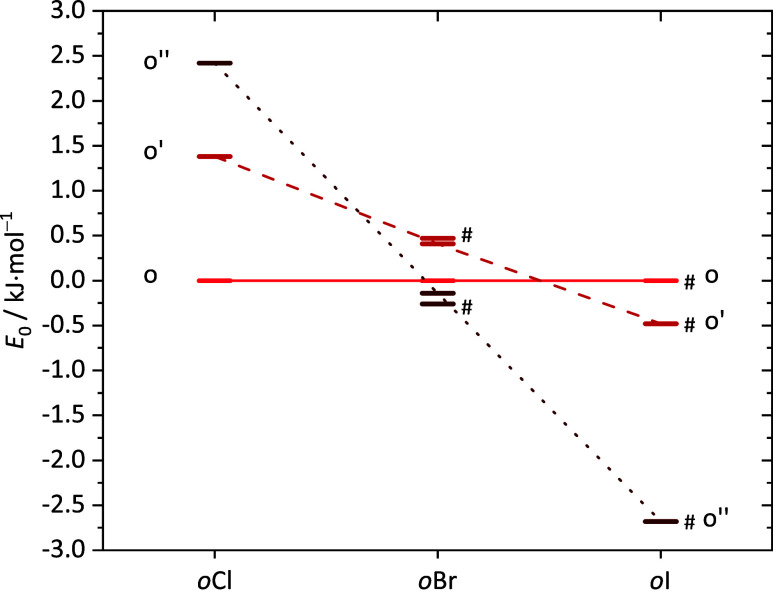
Energetic order of the *o*XT complexes at the DLPNO–CCSD(T)/aug-cc-pVQZ electronic structure level for the B3LYP-D3(BJ)/def2-QZVP-optimized structures with added B3LYP-D3(BJ)/def2-QZVP ZPVE. Electronic structure calculations employing a pseudopotential (aug-cc-pVQZ-PP) are marked with a #.

Interestingly, the very subtle preference switch from o to o″ with increasing size and nuclear charge of the halogen predicted in the B3LYP treatment is strongly amplified when the DFT electronic energy is replaced by the DLPNO–CCSD(T) energy, while keeping the optimized DFT geometry and zero point vibrational energy (Figure 13). The energy gap between o and o″ in Cl is predicted to be inverted for I, with the intermediate Br being close to the crossover. For Br, where calculations with and without pseudopotential were carried out, the influence of the pseudopotential remained small. For I, this test was not feasible, leaving open whether a calculation without the pseudopotential and thus without any included relativistic effects would provide a different trend. With this reservation in mind, there is a strong advantage for o″ at the CCSD(T) wave function level, much larger than at the DFT level. Such a pronounced structural switch, corresponding to torsion of the aromatic unit around the hydrogen bond axis to optimize the halogen interaction, would likely manifest itself in the vibrational spectrum, but as discussed above, it is not evident in the experimental spectra, which suggest a uniform conformation for all three halogens. Our tentative conclusion is that for *o*IT, either the global minimum DFT structures for o/o″ or their relative CCSD(T) energy or both are incorrect. The experimental spectra are more in line with a consistent assignment for all benzyl alcohols, including *o*IT. Structural spectroscopy could shed more light onto this discrepancy.

Therefore, the following discussion concentrates on chlorination effects in the complexation of TEMPO with benzyl alcohol, where experimental and theoretical spectra are in good agreement and the o″ structure is not energetically competitive. It is instructive to compare the highest level energy results for the subtle competition between t and o coordinations in the three investigated systems. This is done in Table 1. For BT, the two coordination variants are more or less isoenergetic. They also require the same amount of monomer deformation. Consequently, the interaction energy is very similar but o has a larger dispersion energy contribution, which is qualitatively expected and also correlates with a reduced downshift of the OH stretching fundamental due to competition with hydrogen bonding. Some of the hydrogen bond strength is sacrificed for a better fit of the two rings. A larger dispersion energy and reduced downshift are also predicted for the chlorinated species in the o conformation, in agreement with expectation and the experimental observation for the wavenumber sequence. *para*-Chlorination destabilizes the o isomer somewhat, largely due to a higher deformation energy. This can be explained by the closer proximity of the Cl atom to the radical ring. *ortho*-Chlorination has the opposite effect of stabilizing the o conformation, both in deformation energy and in interaction energy, turning it into the global minimum structure. This is in good agreement with the experimental findings. Inspection of the NCI plots (SI Figures S8 and S11) suggests that while the o conformation allows for an optimized interaction of one methyl group with the aromatic ring, the t conformation leads to less specific stacking of the two molecules.

**Table 1:** Energetic Comparison between the Two Most Stable Complex Conformations for BT, *p*ClT, and *o*ClT^a^

	*E*_el_	*E*_0_	*E*_el_ (CC)	*E*_0_ (CC)	*E*_geom_^b^	*E*_int_^c^	*E*_disp_^d^	*E*_disp_/*E*_int_
BT t	0	0	0	0	4.72	–46.8	–35.4	0.76
BT o	0.49	0.19	0.11	–0.20	4.84	–46.8	–37.3	0.80
o–t					0.12	0	–1.9	
*p*ClT t	0	0	0	0	2.09	–49.8	–37.0	0.74
*p*ClT o	0.92	0.63	0.60	0.30	2.85	–49.9	–39.2	0.79
o–t					0.76	–0.1	–2.2	
*o*ClT t	0	0	0	0	7.61	–47.8	–36.5	0.76
*o*ClT o	–1.65	–1.45	–1.71	–1.51	7.16	–49.1	–38.0	0.78
o–t					–0.45	–1.3	–1.5	

a Listed are the electronic energy *E*_el_, the zero-point corrected energy *E*_0_ at the B3LYP-D3(BJ)/def2-QZVP level, the electronic energy *E*_el_ (CC) at the DLPNO–CCSD(T)/aug-cc-pVQZ level, as well as the zero-point corrected energy combining CCSD(T) electronic energy and B3LYP/QZVP zero-point energy *E*_0_ (CC), all relative to the t isomer. Additionally, the geometry preparation or deformation energy *E*_geom_ relative to the global minima of the constituents, the interaction energy *E*_int_ at the CCSD(T) level, and the dispersion energy contribution *E*_disp_ determined using LED analysis are shown. All energies are given in kJ mol^–1^.

b *E*_geom_: energy required to distort the monomers to the structure found in the complexes.

c *E*_int_: interaction energy of such distorted monomers in the complex structure.

d *E*_disp_: contribution to *E*_int_ from terms which can be attributed to London dispersion.

So far, the discussion has focused on energetical effects > 0.5 kJ mol^–1^. On a more subtle scale, Table 1 reveals that the o/t energy sequence for the parent complex BT is inverted at the CCSD(T) level, compared to the experimental finding as well as to the DFT prediction. A correction as subtle as 0.3 kJ mol^–1^ would remove this discrepancy. It may arise from neglected aspects such as reoptimization of the structures at the CCSD(T) level, using CCSD(T) instead of DFT for the harmonic zero-point energy, or anharmonic corrections to the zero-point energy and infrared intensity. In the spirit of qualitative (conformer sequence) benchmarking, the experimental energy ranking for the two BT conformations thus remains challenging for high levels of electronic structure and nuclear motion treatment even without halogenation effects.

## 4 Conclusions and Outlook

Multiexperimental spectroscopic approaches^52^ are valuable for mechanistic studies,^53^ in experimental benchmarks for quantum chemistry,^54^ and whenever interesting new phenomena are explored.^17^ The present work prepares the groundwork for how vibrational spectroscopy can contribute to a better understanding of aminoxyl radical–solvent interactions, which are of interest in dynamic nuclear polarization, biological structure determination, and chemical synthesis. By combining a simple stable aminoxyl radical with an aromatic protic solvent (benzyl alcohol), the interplay between hydrogen bonding and dispersion forces^52^ has been uncovered. It can be controlled in subtle ways by halogenation of the aromatic ring. *para-*Chlorination favors one face of the radical, while *ortho*-chlorination favors the other. It would be interesting to combine the two diverging influences by studying 2,4-dichlorobenzyl alcohol, which is a popular oral antiseptic. A potential deficiency of theoretical approaches when switching from Cl and Br to I has been identified.

The aromatic character of benzyl alcohol should allow for size- and conformationally selective double-resonance spectroscopy, which could shed further light onto the microsolvation process, including the regioselectivity for a second solvent molecule. The electrical dipole moments of the complexes are large enough (about 3–6 D) to encourage rotational spectroscopy as the ultimate test for the structural conclusions drawn in this work, in particular, the apparent absence of a structural change when moving from Br to I. Thus, we expect these systems to be investigated by alternative spectroscopic techniques to clarify the nature of NO radical hydrogen bonding in the presence of dispersion interactions. Note that the radical or spin density character itself is not at all evident in the low-resolution infrared spectra.^12^ This will likely be different in high-resolution rotational spectra due to the magnetic fine structure. The microsolvation IR spectra of TEMPO still provide an excellent testing ground for state-of-the-art QM/MM and continuum solvation models,^55^ which try to describe the essence of the solvation process in a more coarse-grained manner.

In the future, we plan to study TEMPO radicals with secondary functional groups^56^ to see how these chemical modifications affect the solvent interaction on the microsolvation scale, with a particular focus on variations in dynamical nuclear polarization enhancement observed in the condensed phase.^57^

## Data Availability

External data sets: raw vibrational spectra (DOI: 10.25625/ZN8QST) and *xyz* structure files of all presented optimized geometries (DOI: 10.25625/GMZZ6E)

## Supporting Information Available

Experimental and computational details: used ORCA keywords for all calculations; influence of isotope masses on ORCA frequency calculations; NCI analyses for *p*ClT t and o structures, for *o*ClT o, o′, o″, t structures and for *o*IT o, o′, o″ structures; comparison between calculated wavenumbers for *p*XT and *o*XT systems; barriers between most stable complexes of BT, *p*ClT, and *o*ClT including transition state structures; details for the *o*XT complexes; relative energies, structures, and torsional angles; list of used chemicals; details for measurement conditions of shown spectra; overview about the experimental and calculated band positions including their deviation; *xyz* coordinates for all discussed complex structures (PDF)
