# Supplementary material for: OH-Detected Aromatic Microsolvation of an Organic NO Radical: Halogenation Controls the Solvation Side
Source: J Phys Chem A. 2025 Jan 30;129(6):1648–58. doi: 10.1021/acs.jpca.4c07744 (PMC11831666; doi:10.1021/acs.jpca.4c07744)
Supplement: Supplementary file 1 — jp4c07744_si_001.pdf [file jp4c07744_si_001.pdf]

Supporting Information:

OH-detected Aromatic Microsolvation of an Organic NO Radical: Halogenation Controls the Solvation Side

Elisabeth Sennert,† Giovanni Bistoni,‡ and Martin A. Suhm*,†

†*Institute of Physical Chemistry, University of Göttingen, Tammannstrasse 6, 37077, Göttingen, Germany*

‡*Dipartmento di Chimica, Biologia e Biotecnologie, Università Degli Studi Di Perugia, Via Elce di sotto 8, 06123, Perugia, Italy*

E-mail: msuhm@gwdg.de

Contents

List of Figures **S2**

List of Tables **S2**

1 Theoretical results **S4**

1.1 Keywords S4

1.2 Influence of isotope masses on ORCA frequency calculations S4

1.3 TEMPO with benzyl alcohol S6

1.3.1 Transition states S6

1.4 TEMPO with *para*-halogenated benzyl alcohols S8

1.4.1 NCI plots S8

1.4.2 Transition states S9

1.4.3 Comparison between calculated wavenumbers for BT, *p*ClT, *p*BrT, *p*IT S11

1.5 TEMPO with *ortho*-halogenated benzyl alcohols S12

1.5.1 NCI plots S12

1.5.2 Transition states S16

1.5.3 More detailed analysis of the *o*XT o conformers S18

1.5.4 Comparison between calculated wavenumbers for BT, *o*ClT, *o*BrT, *o*IT S21

2 Experimental results **S22**

2.1 Used chemicals S22

2.2 Measurement conditions S22

2.3 Experimental and calculated band positions S23

3 Coordinates **S25**

3.1 BT S25

3.2 *p*ClT S30

3.3 *o*ClT S34

References **S40**

List of Figures

S1 BT transition state energies S6

S2 Transition state structures for the BT complexes S7

S3 NCI analysis for *p*ClT t S8

S4 NCI analysis for *p*ClT o S8

S5 *p*ClT transition state energies S9

S6 Transition state structures for the *p*ClT complexes S10

S7 Comparison between the calculated band positions of BT, *p*ClT, *p*BrT, *p*IT o and t complexes S11

S8 NCI analysis for *o*ClT o S12

S9 NCI analysis for *o*ClT o' S12

S10 NCI analysis for *o*ClT o" S13

S11 NCI analysis for *o*ClT t S13

S12 NCI analysis for *o*IT o S14

S13 NCI analysis for *o*IT o' S14

S14 NCI analysis for *o*IT o" S15

S15 *o*ClT transition state energies S16

S16 Transition state structures for the *o*ClT complexes S17

S17 Structures for the *o*ClT o complexes S18

S18 Computed relative conformation energies for *o*X S19

S19 Comparison between the calculated band positions of BT, *o*ClT, *o*BrT, *o*IT o and t complexes S21

List of Tables

S1 Applied ORCA Keywords S4

S2 Influence of isotope masses on frequency calculation in ORCA S5

S3 Torsional angles of different *o*XT o conformers S19

S4 Comparison of the DLPNO-CCSD(T) calculation results for o *o*BrT with different ORCA sub-versions S20
S5 Used chemicals S22
S6 Measurement conditions S22
S7 Comparison between experimental and calculated band positions S24
S8 xyz coordinates for BT t S26
S9 xyz coordinates for BT o S27
S10 xyz coordinates for BT o' S28
S11 xyz coordinates for BT p S29
S12 xyz coordinates for *p*ClT t S31
S13 xyz coordinates for *p*ClT o S32
S14 xyz coordinates for *p*ClT p S33
S15 xyz coordinates for *o*ClT o S35
S16 xyz coordinates for *o*ClT t S36
S17 xyz coordinates for *o*ClT o' S37
S18 xyz coordinates for *o*ClT t' S38
S19 xyz coordinates for *o*ClT o" S39

1 Theoretical results

1.1 Keywords

Tab. S1: Applied keywords in ORCA 5.0.3[S1] for electronic structure optimizations (superscript ES), single point calculations (SP), reaction path optimizations (RP) and transition state searches (TS). For calculations including a radical species the multiplicity was set to 2. Note that transition state calculations are not carried out with the largest basis set, because they are only used to qualitatively assess the conformational isomerization profiles.

Level of approximation	Applied keywords
B97-3c^{ES}	B97-3c abc Opt
B3LYP-D3(BJ)/def2-TZVP^{ES}	B3LYP D3BJ def2-TZVP abc defgrid3 UseSym VERYTIGHTSCF TIGHTOPT FREQ ECP for iodine: def2-ECP
B3LYP-D3(BJ)/def2-QZVP^{ES}	B3LYP D3BJ def2-QZVP abc defgrid3 UseSym VERYTIGHTSCF TIGHTOPT FREQ ECP for iodine: def2-ECP
DLPNO-CCSD(T)/aug-cc-pVQZ^{SP}	UHF DLPNO-CCSD(T) TightPNO aug-cc-pVQZ aug-cc-pVQZ/C TIGHTSCF additional for structures including bromine[1] or iodine: Basis sets: aug-cc-pVQZ-PP aug-cc-pVQZ-PP/C ECP: SK-MCDHF-RSC
NEB-CI/B97-3c^{RP}	NEB-CI B97-3c abc
TS-B97-3c^{TS}	B97-3c abc defgrid3 UseSym OptTS VERYTIGHTSCF TIGHTOPT FREQ
TS-B3LYP-D3(BJ)/def2-TZVP^{TS}	B3LYP D3BJ def2-TZVP abc defgrid3 UseSym OptTS VERYTIGHTSCF TIGHTOPT FREQ

[1] the DLPNO-CCSD(T) calculations including bromine were carried out using ORCA 5.0.4[S1] with the same keywords, for details see Tab. S4

1.2 Influence of isotope masses on ORCA frequency calculations

The choice of masses in the harmonic frequency calculations can influence the results - Tab. S2 exemplifies the influence of the used atom masses on the calculated OH stretching frequencies and IR intensities in ORCA 5.0.3[S1]. The calculated OH stretching wavenumbers and IR

intensities of the t conformation of the *p*XT complexes are shown. For the purposes of this work, the used masses have a negligibly small influence on the wavenumber and on the IR intensity. For OH wavenumbers, the dominant effect is the mass used for H. This work uses the default ORCA option for all calculations.

Tab. S2: Comparison between calculated OH stretching wavenumbers and intensities using different isotope mass options in ORCA 5.0.3[S1] for the atoms in the t conformation of the *p*XT complexes at B3LYP-D3(BJ)/def2-TZVP level. The default option uses an abundance weighted isotope mass which is not the most logical choice in particular for H, while the keyword "Mass2016" uses the mass of the most abundant isotopes. The mass change in H from the default ($1.008\,00\,\mathrm{g\,mol^{-1}}$) to the Mass2016 value ($1.007\,83\,\mathrm{g\,mol^{-1}}$) has a small, but significantly larger effect on OH than the halogen isotope mass due to the local mode character.

**Isotope**	**ORCA option**	M_{Cl} $\mathrm{g\,mol^{-1}}$	M_{H} $\mathrm{g\,mol^{-1}}$	ω_{OH} $\mathrm{cm^{-1}}$	I $\mathrm{km\,mol^{-1}}$
$^{35/37}Cl$	default	35.45300	1.00800	3536.67	634
^{35}Cl	Mass2016	34.96885	1.00783	3536.99	634
^{37}Cl	M=36.96590	36.96590	1.00800	3536.67	634
$^{79/81}Br$	default	79.90000	1.00800	3534.85	627
^{79}Br	Mass2016	78.91834	1.00783	3535.16	627
^{81}Br	M=80.91690	80.91690	1.00800	3534.85	627
^{127}I	default	126.90000	1.00800	3533.75	629
^{127}I	Mass2016	126.90447	1.00783	3534.06	629

1.3 TEMPO with benzyl alcohol

1.3.1 Transition states

In Fig. S1 the height of the barriers between the four most stable BT complexes at B3LYP-D3(BJ)/def2-TZVP level is shown. The structures and imaginary wavenumbers of the transition states can be found in Fig. S2.

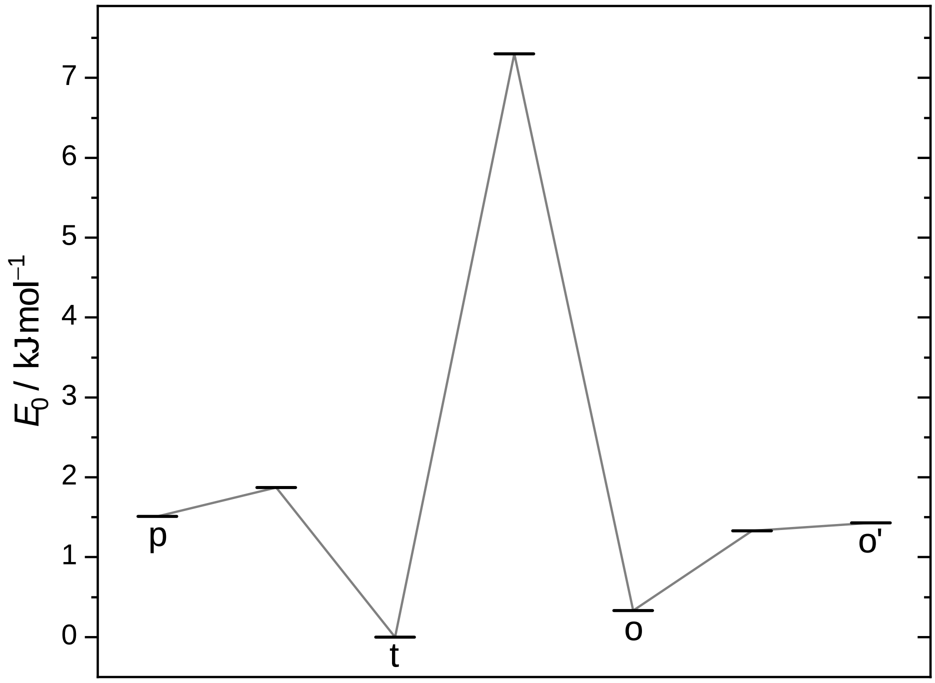

Fig. S1: BT complex energies and calculated transition state energies at B3LYP-D3(BJ)/def2-TZVP level with ZPVE.

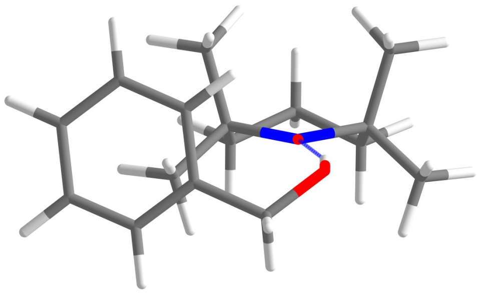

(a) BT o to t; imaginary wavenumber $25.65\,\mathrm{cm}^{-1}$

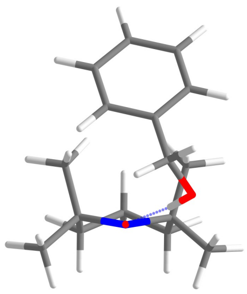

(b) BT p to t; imaginary wavenumber $19.49\,\mathrm{cm}^{-1}$

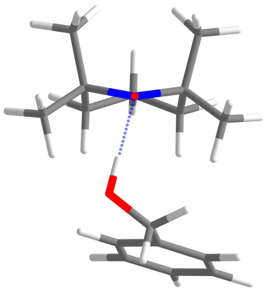

(c) BT o' to o; imaginary wavenumber $12.52\,\mathrm{cm}^{-1}$

Fig. S2: Transition states for the BT complexes o to t (a), p to t (b) and o' to o (c) including the imaginary harmonic wavenumber, calculated at B3LYP-D3(BJ)/def2-TZVP level. The TEMPO conformation is conserved along these paths. Depending on the pdf reader, the imaginary normal coordinate may be animated.

1.4 TEMPO with *para*-halogenated benzyl alcohols

1.4.1 NCI plots

In Fig. S3 and Fig. S4 NCI[S2] analyses, with a reduced density gradient (RDG) value of 0.5 au and the blue-green-red (attractive, van der Waals, repulsive) color scheme ranging from -0.04 to 0.02 au, of the *p*ClT t and o complex interactions are shown. The visualization was done using MultiWFN[S3] and VMD[S4]. Both complexes show attractive interactions between the aromatic ring and one of the TEMPO methyl groups.

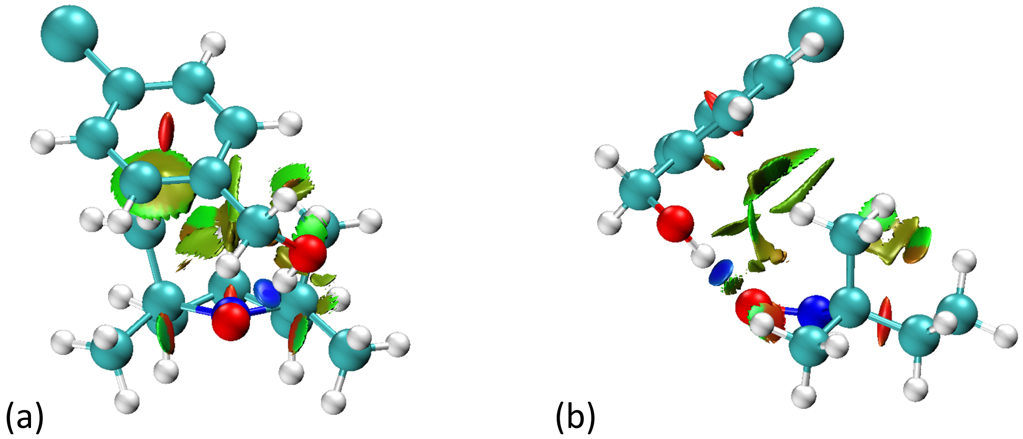

Fig. S3: NCI analysis for *p*ClT t. Structure shown in front view (a) and in side view (b).

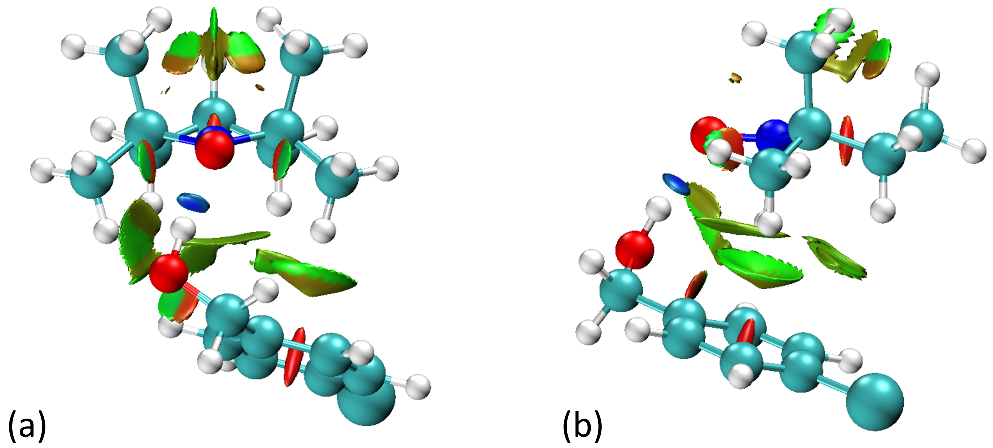

Fig. S4: NCI analysis for *p*ClT o. Structure shown in front view (a) and in side view (b).

1.4.2 Transition states

In Fig. S5 the height of the barriers between the four most stable *p*ClT complexes at B3LYP-D3(BJ)/def2-TZVP level is shown. The structures and imaginary wavenumbers of the transition states can be found in Fig. S6.

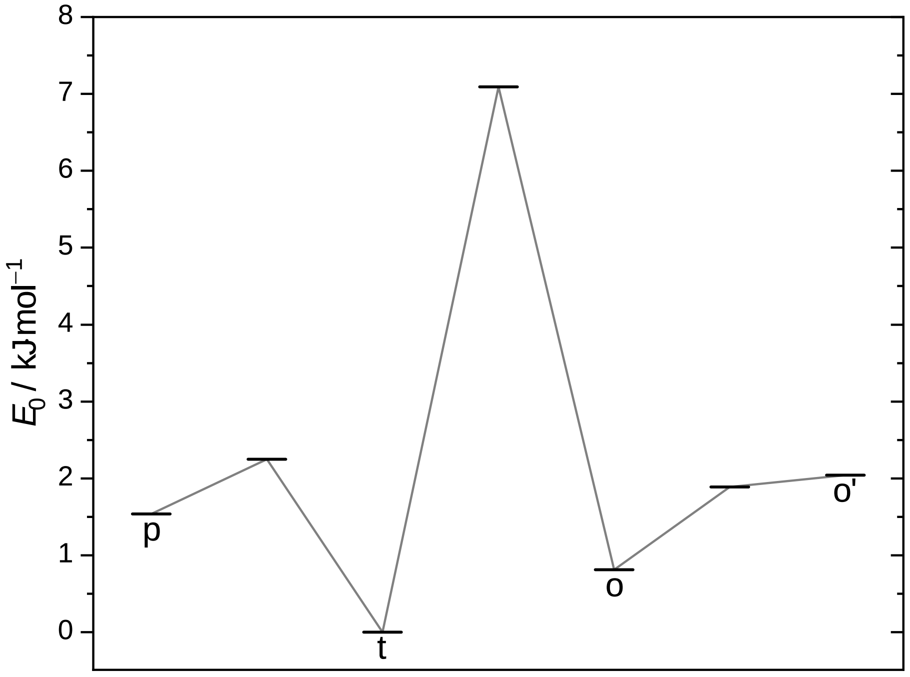

Fig. S5: *p*ClT complex energies and calculated transition state energies at B3LYP-D3(BJ)/def2-TZVP level with ZPVE.

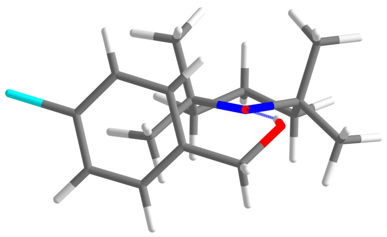

(a) pClT o to t; imaginary wavenumber $25.95\,\mathrm{cm}^{-1}$

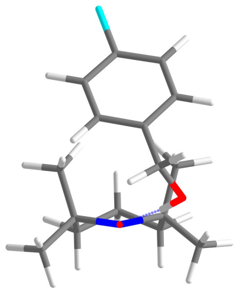

(b) pClT p to t; imaginary wavenumber $18.18\,\mathrm{cm}^{-1}$

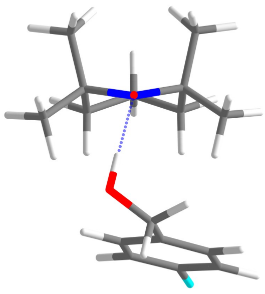

(c) pClT o' to o; imaginary wavenumber $14.07\,\mathrm{cm}^{-1}$

Fig. S6: Transition states for the pClT complexes o to t (a), p to t (b) and o' to o (c) including the imaginary harmonic wavenumber, calculated at B3LYP-D3(BJ)/def2-TZVP level. The TEMPO conformation is conserved along these paths. Depending on the pdf reader, the imaginary normal coordinate may be animated.

1.4.3 Comparison between calculated wavenumbers for BT, *p*ClT, *p*BrT, *p*IT

A comparison between the calculated band positions of the BT, *p*ClT, *p*BrT, *p*IT o and t complexes is shown in Fig. S7.

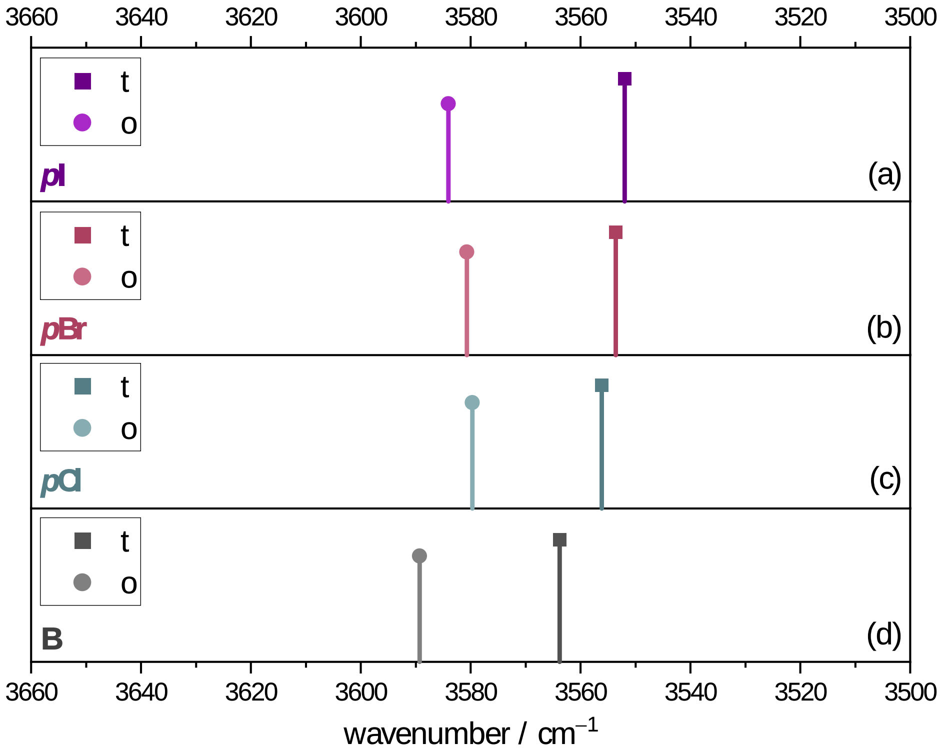

Fig. S7: Comparison between the calculated harmonic wavenumbers and intensities for BT, *p*ClT, *p*BrT, *p*IT o and t complexes, calculated at B3LYP-D3(BJ)/def2-QZVP level.

1.5 TEMPO with *ortho*-halogenated benzyl alcohols

1.5.1 NCI plots

***o*ClT complexes** In Fig. S8, Fig. S9, Fig. S10 and Fig. S11 NCI[S2] analyses, with a reduced density gradient (RDG) value of 0.5 au and the blue-green-red (attractive, van der Waals, repulsive) color scheme ranging from -0.04 to 0.02 au, of the *o*ClT o, o', o" and t complex interactions are shown. The visualization was done using MultiWFN[S3] and VMD[S4].

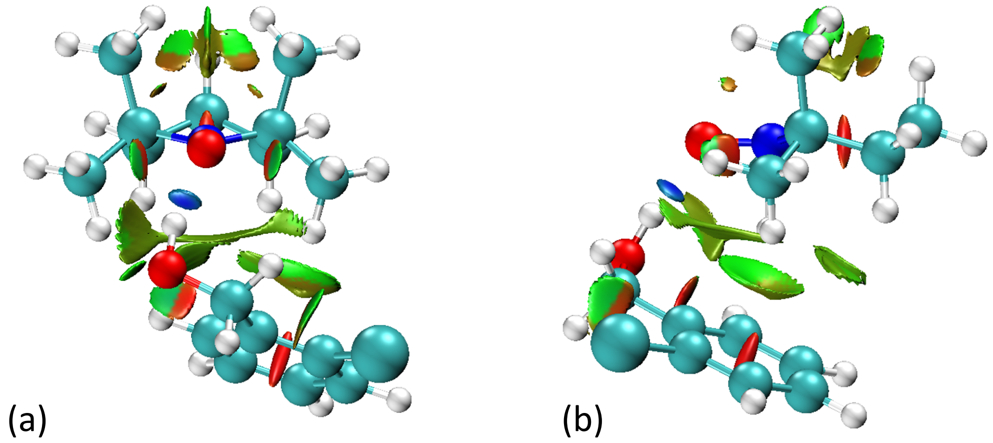

Fig. S8: NCI analysis for *o*ClT o. Structure shown in front view (a) and in side view (b).

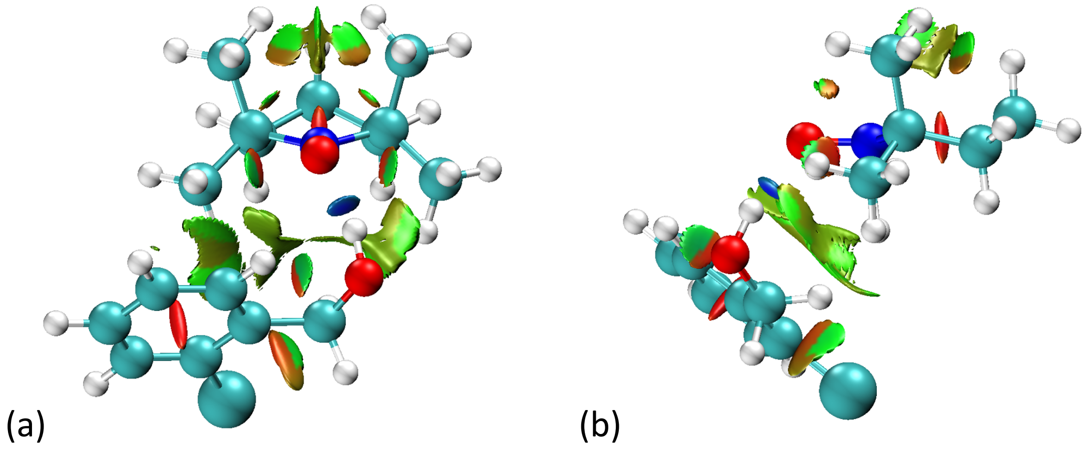

Fig. S9: NCI analysis for *o*ClT o'. Structure shown in front view (a) and in side view (b).

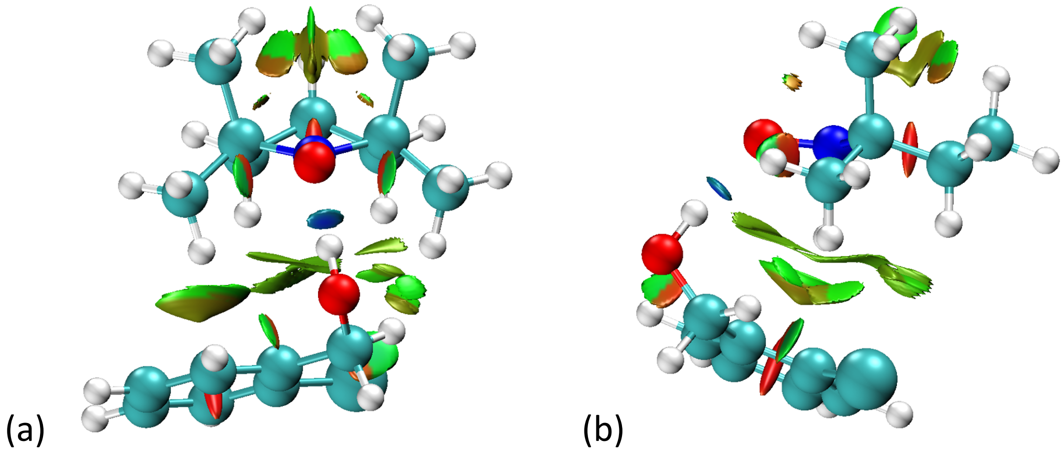

Fig. S10: NCI analysis for *o*ClT o”. Structure shown in front view (a) and in side view (b).

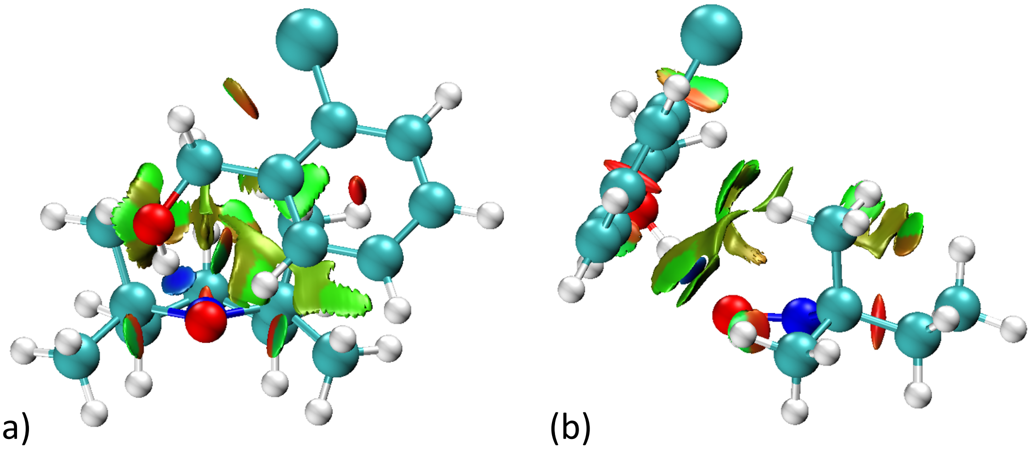

Fig. S11: NCI analysis for *o*ClT t. Structure shown in front view (a) and in side view (b). Note the significantly larger angle between the ring planes which helps to rationalize why the t conformation becomes less competitive upon *ortho*-chlorination of the alcohol donor.

***o*IT complexes** In Fig. S12, Fig. S13 and Fig. S14 NCI[S2] analyses, with a reduced density gradient (RDG) value of 0.5 au and the blue-green-red (attractive, van der Waals, repulsive) color scheme ranging from -0.04 to 0.02 au, of the *o*IT o, o' and o" complex interactions are shown. The visualization was done using MultiWFN[S3] and VMD[S4]. Comparing the NCI analyses of the *o*ClT complexes with the NCI analyses of the *o*IT complexes, the results look very similar.

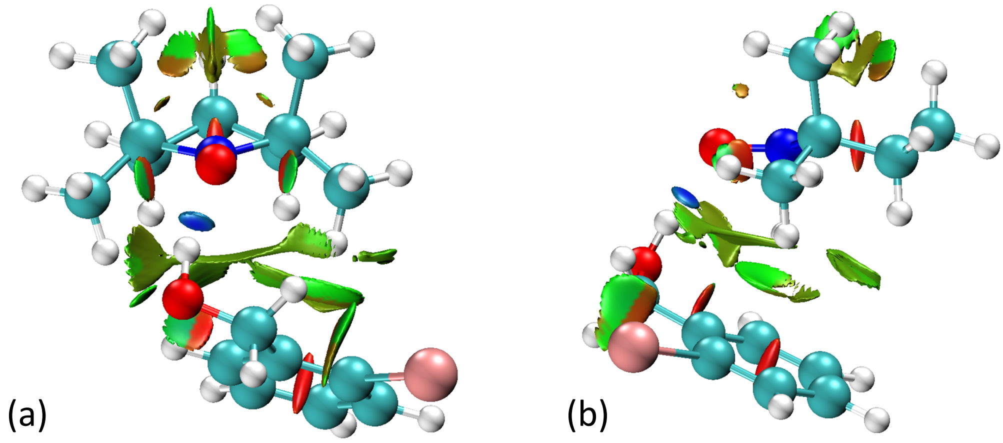

Fig. S12: NCI analysis for *o*IT o. Structure shown in front view (a) and in side view (b).

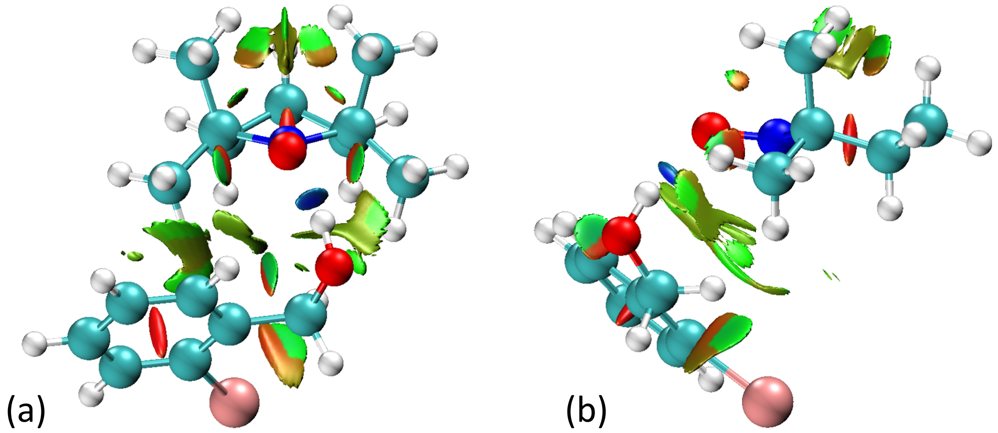

Fig. S13: NCI analysis for *o*IT o'. Structure shown in front view (a) and in side view (b).

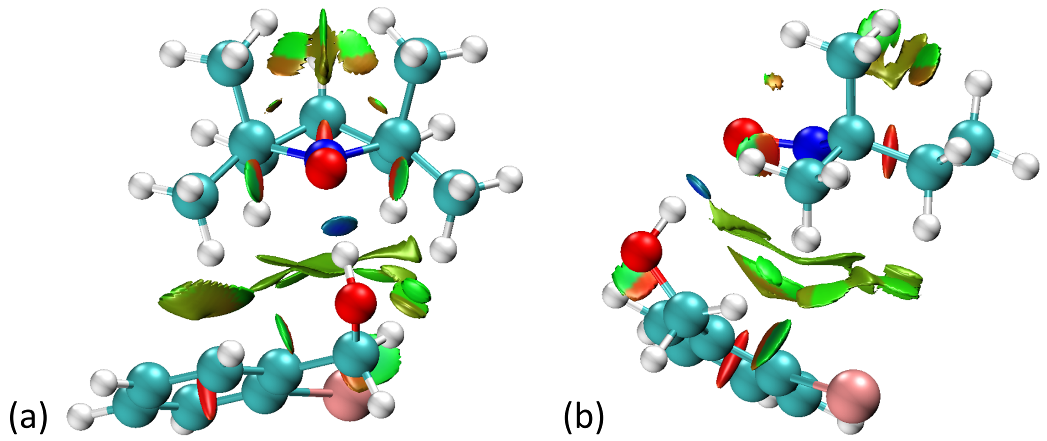

Fig. S14: NCI analysis for *o*IT o". Structure shown in front view (a) and in side view (b). Compared to Fig. S10 there seems to be a slightly stronger interaction of the halogen with the TEMPO which could explain the increased energetic stability Fig. S18.

1.5.2 Transition states

In Fig. S15 the height of the barriers between the four most stable *o*ClT complexes at B3LYP-D3(BJ)/def2-TZVP level is shown. The structures and imaginary wavenumbers of the transition states can be found in Fig. S16.

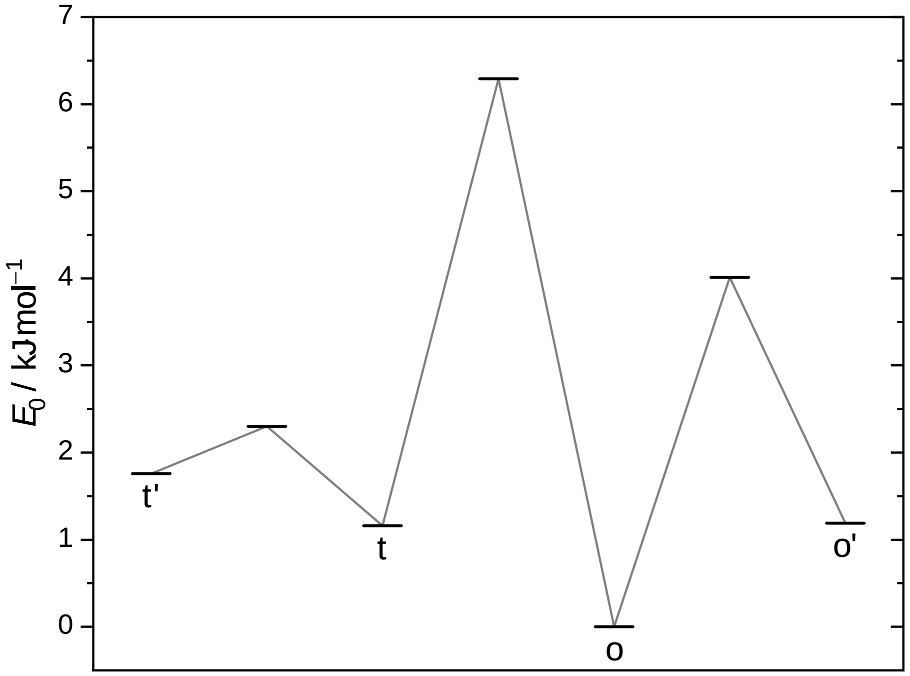

Fig. S15: *o*ClT complex energies and calculated transition state energies at B3LYP-D3(BJ)/def2-TZVP level with ZPVE.

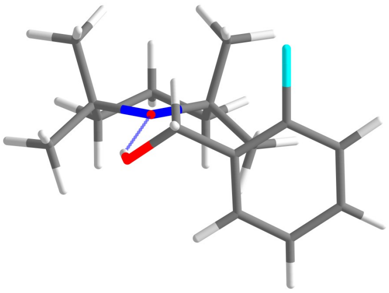

(a) oClT t to o; imaginary wavenumber $16.58\,\mathrm{cm}^{-1}$

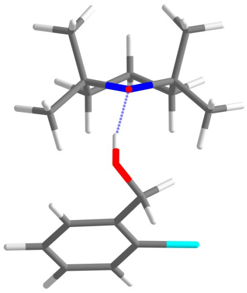

(b) oClT o' to o; imaginary wavenumber $15.31\,\mathrm{cm}^{-1}$

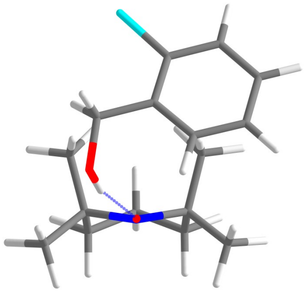

(c) oClT t' to t; imaginary wavenumber $10.63\,\mathrm{cm}^{-1}$

Fig. S16: Transition states for the oClT complexes t to o (a), o' to o (b) and t' to t (c) including the imaginary harmonic wavenumber, calculated at B3LYP-D3(BJ)/def2-TZVP level. The TEMPO conformation is conserved along these paths. Depending on the pdf reader, the imaginary normal coordinate may be animated.

1.5.3 More detailed analysis of the *o*XT o conformers

The optimized structures for the three *o*ClT o conformers are shown in Fig. S17. In Fig. S18 the relative energies (referred to *o*XT o) of t and o” with and without ZPVE are given. A comparison between the torsional angles of the *o*XT o conformers is shown in Tab. S3.

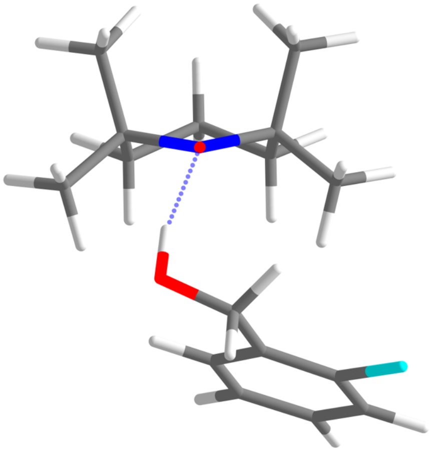

(a) *o*ClT o

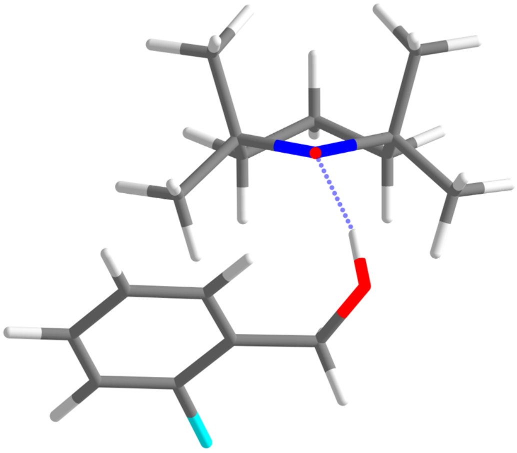

(b) *o*ClT o’

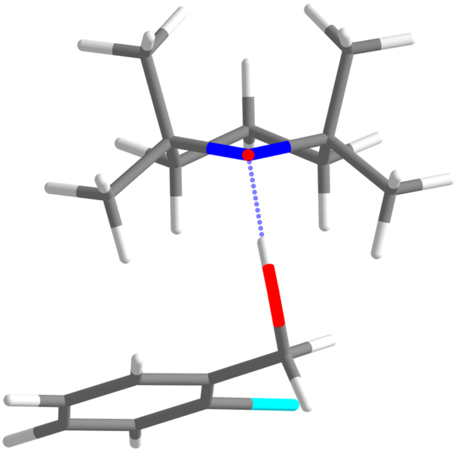

(c) *o*ClT o”

Fig. S17: Optimized structures for the *o*ClT complexes o (a), o’ (b) and o” (c), calculated at B3LYP-D3(BJ)/def2-QZVP level.

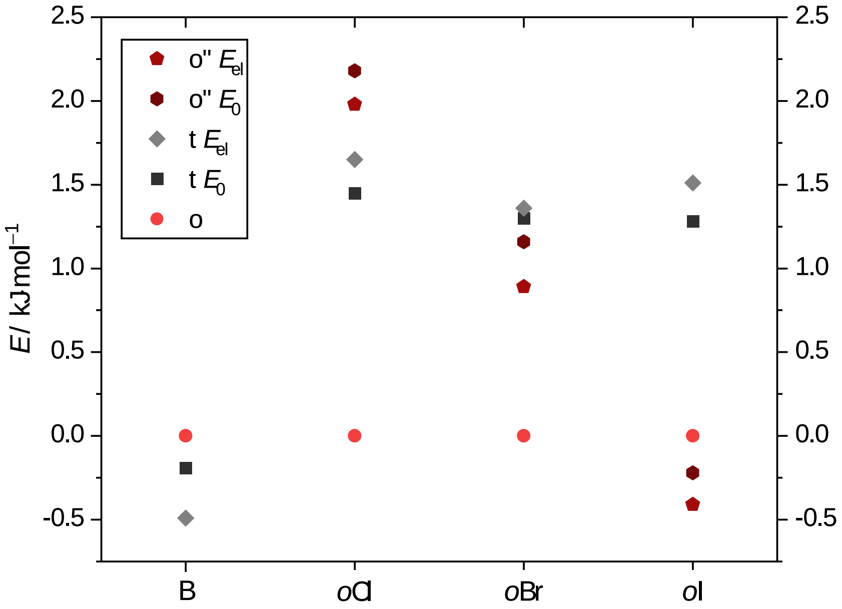

Fig. S18: Computed relative energies of the main conformers for B, *o*Cl, *o*B, *o*I radical complexes, calculated at B3LYP-D3(BJ)/def2-QZVP level with (E_0) and without (E_{el}) ZPVE. The ZPVE correction is seen to have only a minor and non-qualitative effect on the halogenation trend.

Tab. S3: Two torsional angles which are helpful to differentiate between the different *o*XT o conformers. The angles are determined using the optimized B3LYP-D3(BJ)/def2-QZVP structures or their mirror image, whichever generates a positive value. C(X)C(ar)-ON describes the relative orientation of the aromatic CC bond connecting the CX bond to the methylene substituent, and the ON bond. C(ar)C-ON describes the relative orientation of the CC bond connecting the aromatic ring to the methylene group, and the ON bond. The three open coordination variants have comparable angles across the different halogens and are therefore named the same for Cl, Br and I, but their predicted energetical sequence changes from Br to I, see fig. 11 of the main publication. The corresponding C(ar)C-ON angle for the o *p*XT structure (last column) is similar to that of *o*XT, justifying the same naming convention.

System	Conformer	*o*XT C(X)C(ar)-ON	*o*XT C(ar)C-ON	*p*XT C(ar)C-ON
*o*ClT	o	79.9	13.6	24.6
	o’	29.5	104.6	
	o”	0.7	61.4	
*o*BrT	o	77.6	11.2	24.1
	o’	28.7	103.0	
	o”	1.3	63.9	
*o*IT	o	77.7	11.5	23.6
	o’	24.4	96.9	
	o”	5.9	69.8	

In the main document fig. 13 the results of DLPNO-CCSD(T) calculations for the *o*XT

o dimers are shown. For calculating the electronic energy of the *o*BrT o conformers ORCA version 5.0.4 was used. To ensure that this sub-version change is not influencing the results, the calculation for *o*BrT o was carried out with versions 5.0.3 and 5.0.4. A comparison between the, for this publication, most relevant values is shown in Tab. S4.

Tab. S4: Comparison of the DLPNO-CCSD(T) calculation results for o *o*BrT with different ORCA sub-versions. The absolute calculated electronic energy E_{el}, the interaction energy E_{int} and the dispersion energy contribution E_{disp} determined using LED analysis are shown. All energies are given in kJ/mol.

ORCA version	E_{el}	E_{int}	E_{disp}	E_{disp} / E_{int}
5.0.3	−8930688.17308	−51.41	−38.35	0.75
5.0.4	−8930688.17308	−51.41	−38.35	0.75

1.5.4 Comparison between calculated wavenumbers for BT, *o*ClT, *o*BrT, *o*IT

A comparison between the calculated band positions of the BT, *o*ClT, *o*BrT, *o*IT o and t isomers is shown in Fig. S19. If the harmonically predicted energetical sequence switch from Br to I is correct, it should manifest itself in an unusually large splitting between the t and o signals in the IR spectrum.

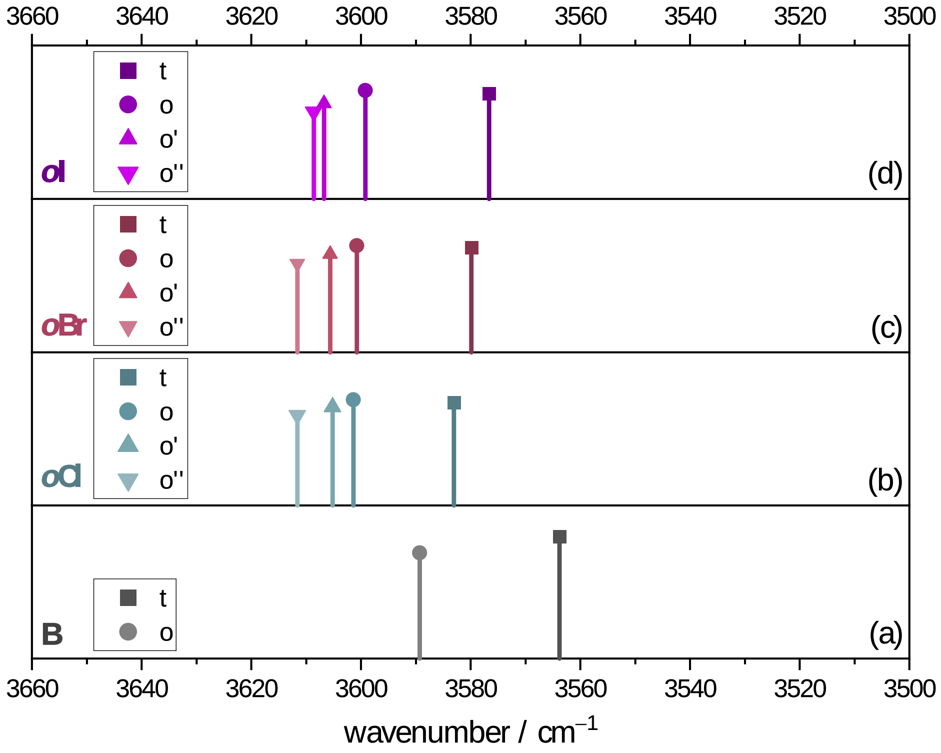

Fig. S19: Comparison between the calculated harmonic wavenumbers and intensities for BT, *o*ClT, *o*BrT, *o*IT o and t complexes, calculated at scaled harmonic B3LYP-D3(BJ)/def2-QZVP level. Comparison to the experimental wavenumber trends suggests that the three halogen variants of the alcohol share the same conformational preference relative to TEMPO, with o being more consistent with experiment than o' or o".

2 Experimental results

2.1 Used chemicals

Tab. S5: List of used chemicals, CAS number, supplier and purity.

Name	CAS	Supplier	Purity
Helium	7440-59-7	Nippon Gases, Düsseldorf	$\geq$ 99.996 %
2,2,6,6-Tetramethyl-1-oxylpiperidine	2564-83-2	BLD Pharmtech, Kaiserslautern	$\geq$ 97 %
Benzyl alcohol	100-51-6	Sigma-Aldrich, Taufkirchen	$\geq$ 99.0 %
(4-Chlorophenyl)methanol	873-76-7	BLD Pharmtech, Kaiserslautern	$\geq$ 98 %
(4-Bromophenyl)methanol	873-75-6	BLD Pharmtech, Kaiserslautern	$\geq$ 98 %
(4-Iodophenyl)methanol	18282-51-4	BLD Pharmtech, Kaiserslautern	$\geq$ 98 %
2-Chlorobenzyl alcohol	17849-38-6	BLD Pharmtech, Kaiserslautern	$\geq$ 98 %
2-Bromobenzyl alcohol	18982-54-2	BLD Pharmtech, Kaiserslautern	$\geq$ 98 %
2-Iodobenzyl alcohol	5159-41-1	BLD Pharmtech, Kaiserslautern	$\geq$ 98 %

2.2 Measurement conditions

Tab. S6: Details for the measurement conditions of the shown spectra in the main publication including the temperature of the first substance chamber T_1, the temperature of the second substance chamber T_2, the temperature of the nozzle T_{Nozzle} and the number of averaged scans #. Raw vibrational spectra are published as a separate dataset (DOI:10.25625/ZN8QST).

Substance 1	T_1 / °C	Substance 2	T_2 / °C	T_{Nozzle} / °C	#	Figure
TEMPO	35	Benzyl alcohol	50	70	200	3, 7, 10
-	-	Benzyl alcohol	70	90	313	3[1]
-	-	TEMPO	50	70	56	3[2],5[1]
TEMPO	35	4-Chlorobenzyl alcohol	85	105	226	5, 7
-	-	4-Chlorobenzyl alcohol	80	100	191	5[1,3]
TEMPO	25-35	4-Bromobenzyl alcohol	105	125	275	7
TEMPO	20	4-Iodobenzyl alcohol	115	135	325	7
TEMPO	35	2-Chlorobenzyl alcohol	70	90	425	9, 10
TEMPO	35	2-Bromobenzyl alcohol	90	110	536	10
TEMPO	35	2-Iodobenzyl alcohol	110	130	350	10

1 only CH region shown, intensity multiplied by 0.5 for better comparison
2 only CH region shown, intensity multiplied by 0.4 for better comparison
3 measured by M. Lange, OH region published in the ESI of[S5]

2.3 Experimental and calculated band positions

In Tab. S7 the experimentally observed band positions and assignments to the computed conformations of the alcohol-radical complexes are shown. Scaled harmonic band positions for complexes are also listed and, if applicable, compared to the experiment.

Tab. S7: Experimental band positions ($\tilde{\nu}$), calculated band positions at B3LYP-D3(BJ)/def2-QZVP level (ω_{calc}), scaled band positions ($\omega_{\mathrm{scal}} = \omega_{\mathrm{calc}} \cdot 0.97$), calculated IR intensity (I_{calc}) and difference between experimental and scaled band positions ($\Delta\omega = \omega_{\mathrm{scal}} - \tilde{\nu}$) for alcohol-radical complexes. The deviations between experiment and scaled harmonic theory are very systematic, supporting the proposed assignments and the ability of dispersion-corrected harmonic DFT calculations to capture any differences between different halogen atoms consistently. A distinction between o and o" in the *o*IT case is difficult due to the limited experimental sensitivity and growing impurities due to decomposition at higher temperature, but analogy to Cl and Br suggests an o assignment (alternative assignments marked with *).

**Complex**	**Structure**	$\tilde{\nu}$ $\mathrm{cm^{-1}}$	ω_{calc} $\mathrm{cm^{-1}}$	ω_{scal} $\mathrm{cm^{-1}}$	I_{calc} $\mathrm{km\,mol^{-1}}$	$\Delta\omega$ $\mathrm{cm^{-1}}$
BT	t	3433	3563.8	3456.9	637	+23.9
	o	3454	3589.3	3481.6	550	+27.6
	o'	-	3601.8	3493.7	469	-
	p	-	3601.1	3493.1	626	-
*p*ClT	t	3425	3556.2	3449.5	642	+24.5
	o	3447	3579.7	3472.3	551	+25.3
	p	-	3591.1	3483.4	630	-
*p*BrT	t	3424	3553.6	3447.0	640	+23.0
	o	3445	3580.7	3473.3	536	+28.3
*p*IT	t	3424	3552.0	3445.4	639	+21.4
	o	3445	3584.1	3476.6	508	+31.6
*o*ClT	t	3455	3583.1	3475.6	536	+20.6
	t'	-	3626.5	3517.7	533	-
	o	3471	3601.4	3493.4	551	+22.4
	o'	3471*	3605.2	3497.0	514	+26.0
	o"	3471*	3611.6	3503.3	471	+32.3
*o*BrT	t	3455	3580.0	3472.6	545	+17.6
	o	3471	3600.8	3492.8	535	+21.8
	o'	3471*	3605.6	3497.4	512	+26.4
	o"	3471*	3611.6	3503.3	463	+32.3
*o*IT	t	3455	3576.6	3469.3	548	+14.3
	o	3469	3599.2	3491.2	565	+22.2
	o'	3469*	3606.8	3498.6	496	+29.6
	o"	3469*	3608.6	3500.3	454	+31.3

3 Coordinates

All presented xyz files are also available as a separate dataset (DOI:10.25625/GMZZ6E).

3.1 BT

The xyz files for the four most stable BT complexes (Fig. 2 in the main publication) are listed in Tab. S8, Tab. S9, Tab. S10 and Tab. S11.

Tab. S8: xyz coordinates for BT t at B3LYP-D3(BJ)/def2-QZVP level.

atom	x	y	z
C	-2.64084997415851	-2.57339122995157	-0.28633102454603
C	-3.15938959284440	-1.23266080593151	0.21333948315719
C	-2.05640435843672	-0.18542461930626	0.41673264980535
C	-0.42686726513176	-2.20392077982118	0.93947145442066
C	-1.63579304258258	-3.12332211686177	0.71558956053695
H	-2.18696880849891	-2.47000750411609	-1.27293837665112
H	-3.47025073104697	-3.27220893119177	-0.40125735808269
H	-3.67188255407792	-1.38956120759786	1.16567576003149
H	-3.89416501351824	-0.81279726557434	-0.47454989171235
H	-2.14170428982026	-3.27401945517412	1.67241875371187
H	-1.25881851118023	-4.09717713873268	0.40151359359467
C	-2.59387271476494	0.95723206557498	1.28031743433555
H	-2.85547611558732	0.59905101669168	2.27504850424261
H	-1.85975626764533	1.75040175597765	1.38218765978698
H	-3.48953997798186	1.36314332659321	0.81106381507220
C	-1.55948814728857	0.38177734298689	-0.92122030461480
H	-1.27915541277485	-0.39763585014884	-1.62438145802586
H	-2.34946167546448	0.97780748244174	-1.37743087699059
H	-0.69701478057941	1.02157952942978	-0.75934556763685
C	0.30974028267566	-2.63784458431525	2.20875413636258
H	1.22566965280782	-2.07369635066541	2.35514138838459
H	-0.32291998639992	-2.50432604628406	3.08500561217351
H	0.56505082527111	-3.69363346290325	2.12343822927763
C	0.54087705844457	-2.25480499225228	-0.25381677946128
H	1.34424173059101	-1.53520183680829	-0.12224801982131
H	0.97939650872137	-3.25077665350373	-0.31410066438417
H	0.04333366662246	-2.04813204174047	-1.19756916847276
N	-0.90050418733056	-0.79853810175673	1.14488707670572
O	-0.07000813004623	0.02426748093733	1.66086341209532
C	1.58903043121738	1.26259352615583	-2.79544453464682
C	2.22828898842870	0.97828538239532	-1.59741407388984
C	0.84036130988576	2.42812493708571	-2.93033591335690
H	2.80551256116455	0.07074697969276	-1.48953999535151
C	2.12782732676566	1.84971897176286	-0.51278436260535
C	0.73328818741997	3.30160784323278	-1.85649634542647
H	0.14659921097475	4.20524542781142	-1.94879156534702
C	1.37066773680509	3.00882113261684	-0.65498855349485
H	1.27311744697594	3.68494141372556	0.18454109718381
H	1.67504732836711	0.57693411585878	-3.62742432697120
C	2.84115748691114	1.54079651321412	0.78460765190322
H	3.91687307667898	1.67132204190245	0.64997446298207
H	2.52182446518431	2.25561100308815	1.54996588737038
O	2.66186896194131	0.20716833529005	1.22531170743988
H	1.72164873008476	0.09109191460758	1.44812894033341
H	0.34190351481219	2.64947583224233	-3.86376127412430

Tab. S9: xyz coordinates for BT o at B3LYP-D3(BJ)/def2-QZVP level.

atom	x	y	z
C	-2.87539204994203	-1.64926600181027	-0.58648409129650
C	-1.63845039681796	-2.45741183993544	-0.22127418419388
C	-0.95740343958865	-1.98905779381466	1.07164586179140
C	-1.85702532751029	0.42996606871368	0.50187727046842
C	-2.48479726528446	-0.18800400551012	-0.75460372266408
H	-3.64945857172698	-1.75986695969736	0.17464978535384
H	-3.30334007624156	-2.02772240801213	-1.51553959034008
H	-0.91700963831219	-2.38086378377918	-1.03807387158812
H	-1.87730739642911	-3.51556819843117	-0.10805988296885
H	-1.76496354825870	-0.10738863139511	-1.57205161593631
H	-3.34658571136138	0.42099098998563	-1.03029960336817
C	0.44236244028469	-2.59822917318906	1.15433576954948
H	1.06740199413539	-2.24791008222247	0.33695373987324
H	0.93298795016501	-2.34734231400191	2.08971718050720
H	0.35767419255937	-3.68221695146471	1.08561489226415
C	-1.75820185383726	-2.40532622228911	2.31633847442109
H	-1.32497453020999	-1.94768394970978	3.20330659591000
H	-2.80438077421756	-2.11846856955910	2.25406078563873
H	-1.71261579601292	-3.48817617510332	2.42878701418796
C	-1.16428272578485	1.73828755860547	0.12281179731742
H	-0.73961972334520	2.22590273611030	0.99512507932498
H	-0.36998944613824	1.56043333021674	-0.59561037177683
H	-1.89714670968641	2.40509731312923	-0.33048843547882
C	-2.91023016499640	0.70883216492630	1.58618834586413
H	-2.41919031269180	1.02221524155297	2.50525210046569
H	-3.56562989891470	1.51294266797165	1.25296206373264
H	-3.52784524851475	-0.15893936019926	1.80225087756451
N	-0.81713903802411	-0.49729853299705	1.05420347990102
O	-0.00449483387303	0.00895530458610	1.90053699602807
C	1.56185271148785	2.66379460316152	-2.49407446170342
C	2.16788048591384	2.53262806159750	-1.25117180112232
C	1.15871098800811	1.53288921153273	-3.19545796564130
H	2.45861698300869	3.41832557368433	-0.70028182150087
C	2.38753010788555	1.27519263242892	-0.69244135242218
C	1.37770245317449	0.27451422314850	-2.64704362334406
H	1.07153876470753	-0.61106193494046	-3.18757912898322
C	1.99180510003412	0.14796900272264	-1.40681975852501
H	2.16244734199781	-0.82684489545895	-0.97594978079654
H	1.39214711555133	3.64765301283056	-2.90939165106837
C	3.04906595866475	1.14775888945819	0.66437370908079
H	4.13431993483144	1.17070036171610	0.53918011527177
H	2.78123726395430	2.01989744720874	1.27124246096657
O	2.75746892150281	-0.05469457592526	1.34035522028049
H	1.81758611880295	-0.05284425085667	1.58867686329118
H	0.67803321846442	1.63229110718122	-4.15863933027467

Tab. S10: xyz coordinates for BT o' at B3LYP-D3(BJ)/def2-QZVP level.

atom	x	y	z
C	-2.67716088407309	-0.65206152220211	-0.88916029889963
C	-1.61569529212237	-1.69339103282438	-0.56004872444539
C	-1.13370514809106	-1.63990343465592	0.89664823097966
C	-1.67955240357489	0.93670080134316	0.84933206907773
C	-2.12883117796106	0.74021153689801	-0.60570206698165
H	-3.58928018079829	-0.83363194495595	-0.31811872410206
H	-2.95468397706187	-0.73014311517143	-1.94101328419373
H	-0.75448307943053	-1.53368440648830	-1.21143244765688
H	-1.98277076637798	-2.70202529731170	-0.75505800795797
H	-1.27104590632279	0.91900793215395	-1.25704844414863
H	-2.86993315024652	1.50735202146189	-0.83481830394395
C	0.14755672994504	-2.46065912494842	1.03993330725683
H	0.95261840875106	-2.02920981499169	0.45354697992797
H	0.47022024215866	-2.51503419623278	2.07540718198228
H	-0.04436124771922	-3.47081754861905	0.67941029634079
C	-2.18672181949643	-2.19863042881689	1.86875054796265
H	-3.17388569110644	-1.77417332969233	1.71114907743354
H	-2.26117488993266	-3.27716442564128	1.73231850197948
H	-1.88395254870538	-2.00337815882573	2.89577534756664
C	-0.83899122809153	2.20876105336946	0.95313006159462
H	0.05604457365563	2.12427401327692	0.34451355576774
H	-1.42774642349232	3.04759487276840	0.58306631928755
H	-0.54818472177691	2.40839957195145	1.97998736265257
C	-2.87565894037274	1.05601884036297	1.80840523176688
H	-2.52753479407016	1.03924670797334	2.83950669357565
H	-3.38268942115373	2.00435116610437	1.63230892217227
H	-3.60221147454441	0.26009905245984	1.67479057479873
N	-0.81994333468479	-0.21990876772617	1.25508245860482
O	-0.02543693668838	-0.03035601179561	2.23832570052719
C	1.26041264677955	1.55967675855085	-2.85509620318980
C	1.79751875187227	1.81261830431161	-1.59804620622321
C	1.23450981352353	0.26229255947701	-3.35178321666746
H	1.80538602805145	2.82452120552398	-1.21320600113628
C	2.31562402143782	0.77990250571532	-0.82023853139013
C	1.76132138521486	-0.77366675845577	-2.58771034732433
H	1.75337214745723	-1.78518919884733	-2.97047893134099
C	2.30071685515590	-0.51478386021911	-1.33446377032601
H	2.71073816986494	-1.31638335963564	-0.73799573846647
H	0.85592101821365	2.37322156080891	-3.44151916642820
C	2.90298955186209	1.06052897584739	0.54832375114942
H	3.98197107337730	1.20337619823972	0.45236252402804
H	2.49156295057358	2.00083189336975	0.92989212597478
O	2.72869750408805	0.01033924364883	1.47566500710257
H	1.80222311741344	-0.01011042289255	1.76753108653604
H	0.81116932838240	0.06046013815960	-4.32582221598974

Tab. S11: xyz coordinates for BT p at B3LYP-D3(BJ)/def2-QZVP level.

atom	x	y	z
C	-3.15866642763101	-1.95120123942305	-0.23177545753273
C	-1.87897723101000	-2.72084044642787	0.05967208076312
C	-0.64459066598047	-1.82395807526198	0.22910066421887
C	-2.30485790137968	0.01815071642883	1.15614076240655
C	-3.43305548282928	-0.99422866039905	0.91871658659951
H	-3.07951210125659	-1.41070712026340	-1.17614566078192
H	-3.99250126171321	-2.64519096866725	-0.34379575886254
H	-2.01928639115829	-3.29910922842219	0.97630966842652
H	-1.65999391928096	-3.43640915289521	-0.73345234214700
H	-3.57562126221834	-1.57767854794045	1.83150671486993
H	-4.35452650850688	-0.43381923346076	0.75640558809980
C	0.47523830190294	-2.63412387228282	0.88732035456760
H	1.41847937589652	-2.09726244946581	0.89250276747394
H	0.61380917420296	-3.55709565685826	0.32467193878217
H	0.21272892712795	-2.89234922446601	1.91219609914802
C	-0.15582888782555	-1.28154311191902	-1.12194162680661
H	-0.95075989841514	-0.80535300764001	-1.68976035459499
H	0.23736921890108	-2.10473044422915	-1.71786482323682
H	0.63983100140962	-0.55827491247227	-0.97351118549694
C	-2.47250904996071	0.64646412735937	2.54120078040610
H	-1.72582188852083	1.41445472302400	2.71804122284638
H	-2.37876666277092	-0.10948279461732	3.31954716311428
H	-3.46360941022946	1.09389560889105	2.61065090495775
C	-2.31078933802506	1.12542805437915	0.09087689789986
H	-3.20467131613559	1.73744212229051	0.20902774682653
H	-2.30437888729645	0.72689323383879	-0.92021204199067
H	-1.43540611145125	1.75809374347302	0.21221298947105
N	-0.97785810954405	-0.67565805631708	1.13176994772814
O	0.00170026417799	0.00247069252952	1.59376931021778
C	0.77349126067502	2.42379254454009	-1.94412663530962
C	1.36726809973635	2.28317061812102	-0.69468992448936
C	1.29905996580431	1.75512718741885	-3.04180720126106
H	0.95168040283061	2.79551714512029	0.16279348941944
C	2.48817889565809	1.47575057873118	-0.52450285919084
C	2.42226247856021	0.94842296861681	-2.88423545017866
H	2.83772247850032	0.42798235362180	-3.73622587628094
C	3.01024936898773	0.81163761959930	-1.63481223519543
H	3.87617581960585	0.17513309445731	-1.50982368514573
H	-0.09945857211933	3.05155251931428	-2.05917754616051
C	3.09544267531493	1.26933260323389	0.84183405456109
H	2.75515980381549	2.06147043408004	1.51704230349792
H	4.18243811331886	1.32984404276278	0.78238414969736
O	2.80252211747228	-0.01250513566266	1.37767218486567
H	1.83894356071308	-0.07942658178036	1.48452040965575
H	0.84014231248274	1.86247886766907	-4.01484429857736

3.2 *p*ClT

The xyz files for the three most stable *p*ClT complexes (Fig. 4 in the main publication) are listed in Tab. S12, Tab. S13 and Tab. S14.

Tab. S12: xyz coordinates for *p*ClT t at B3LYP-D3(BJ)/def2-QZVP level.

atom	x	y	z
C	-2.04503168346665	-2.82914227810359	-1.76169373204347
C	-0.62856737226844	-3.23780370506168	-1.38393393664430
C	0.25821205379185	-2.05802395544483	-0.96449006670040
C	-1.19844468986170	-0.56283357424350	-2.59194811409165
C	-1.98573407867590	-1.84015427280470	-2.91695128998865
H	-2.56418371421018	-2.39494808350737	-0.90609085345289
H	-2.61897415190829	-3.70868500302738	-2.05547369700619
H	-0.16763314847135	-3.73422609182812	-2.24140011105529
H	-0.63096224146834	-3.95886747365548	-0.56582415924542
H	-1.51670295676294	-2.33019377109534	-3.77368960943355
H	-2.98597552161623	-1.54222148886648	-3.23293093183196
C	1.72594362158944	-2.48812850593436	-0.99669579313280
H	2.37476387291206	-1.69677173517254	-0.63423066330940
H	1.85081132532957	-3.36505300201559	-0.36227473847135
H	2.03014991954115	-2.74700491723226	-2.00992253430725
C	-0.09235733588565	-1.55953519389505	0.44499631005265
H	-1.15289578209165	-1.35437962426495	0.56185294246471
H	0.18579507491933	-2.31554792053489	1.17854115963875
H	0.45877432465664	-0.65008391970019	0.66342672360155
C	-0.86966175986251	0.17178262259801	-3.89365223997390
H	-0.39512013550269	1.13038356261678	-3.70765883395000
H	-0.20670620897088	-0.42752625753823	-4.51589891364922
H	-1.79481270355305	0.34656193994918	-4.44195810074562
C	-2.00258941105207	0.36766411137888	-1.66962248545046
H	-1.40200439525413	1.22471012383930	-1.37722075581400
H	-2.87867782861864	0.73099211066182	-2.20644209363518
H	-2.34405326185359	-0.13794429448080	-0.77036044917712
N	0.09573732224912	-0.92743682832875	-1.93364749770620
O	0.99714684732028	-0.02248735129087	-1.88669372910116
C	-0.64807041075967	1.60148683132958	2.33753040425098
C	-0.22148036780970	2.26630609890007	1.19825538375722
C	0.27975580081233	0.90911625779098	3.10513605989239
H	-0.93820108285810	2.79843710743045	0.58908330249596
C	1.11800543398142	2.24604415601501	0.81112566166397
C	1.61853149551097	0.87956027315814	2.74967087727530
H	2.32632186079014	0.33034235587887	3.35196762366443
C	2.02564372039452	1.54643824084449	1.59951298971994
H	3.06679913056308	1.50809327721936	1.30770484264368
H	-1.68730356882335	1.61354669135528	2.63007268049793
C	1.56332152387490	2.96977050801626	-0.43946244167425
H	1.52628042334579	4.04825614776018	-0.27327297939834
H	2.60542604391050	2.71051326881972	-0.65284718756554
O	0.73354170181021	2.71723703084299	-1.55761528113408
H	0.80104015860358	1.76948479617783	-1.77027204509657
Cl	-0.25488886982072	0.04266940963858	4.52513003042082

Tab. S13: xyz coordinates for *p*ClT o at B3LYP-D3(BJ)/def2-QZVP level.

atom	x	y	z
C	-1.68906487543483	-2.42382496888945	-1.71308184659475
C	-1.50082751948243	-1.13715578935333	-2.50417155232123
C	-0.03024613840655	-0.78694009724991	-2.76846994907253
C	0.52692715610195	-2.02082874123572	-0.50006514899919
C	-0.97711222178364	-2.29804824774004	-0.37389430107091
H	-1.31370786334588	-3.28132308147362	-2.27384356815208
H	-2.75199249792539	-2.60596674927610	-1.55113237645884
H	-1.95712131682139	-0.31446816746814	-1.94870432682159
H	-2.00997555203541	-1.18851616375454	-3.46729496587996
H	-1.43081215672324	-1.48113005017390	0.19143174037247
H	-1.10079149525688	-3.20166506236923	0.22395661845526
C	0.06885313112888	0.66537837181893	-3.23558225958371
H	1.08460400311994	0.92654171356430	-3.51642874986805
H	-0.57649563663341	0.79820112444294	-4.10311502441101
H	-0.25377473026579	1.35184811137374	-2.45708432663200
C	0.58392049122266	-1.69718584219793	-3.84496853731405
H	0.41799827065490	-2.75196180859493	-3.64457377430947
H	0.13649042848919	-1.46450533777974	-4.81081453813868
H	1.65554921846267	-1.52036125498362	-3.90996278625693
C	1.06260598138028	-1.54416491407965	0.84919903478944
H	2.13468519846070	-1.37632168437982	0.81103348648141
H	0.57788955940781	-0.62144369408496	1.15147863974403
H	0.85188256237029	-2.30554767215070	1.59933976795182
C	1.30257297912414	-3.27373918202566	-0.93783705553417
H	2.33792086233466	-3.01275661419851	-1.14758100903228
H	1.28753785643564	-4.00569680803113	-0.13097134922469
H	0.87727073995432	-3.74130926826039	-1.82162462273774
N	0.75359446044889	-0.92620894956944	-1.49892561250115
O	1.90223377138152	-0.36606364681898	-1.47202623150218
C	-1.37225693757756	1.56874958725740	1.30704869522836
C	-0.28429647199924	2.02767090497099	0.57652988009035
C	-1.19402898227663	1.20942154509480	2.63505586259847
H	-0.40846780591658	2.30273027608516	-0.45945118361322
C	0.97902256406279	2.12364369633435	1.15187195842643
C	0.05125802084741	1.31161760838172	3.23850456725267
H	0.17620542431763	1.02410240751127	4.27149845797227
C	1.12847109316146	1.76542577842328	2.48939130203726
H	2.10508126783708	1.82140432288499	2.95275462393698
H	-2.34901265397420	1.48860210784470	0.85366653116829
C	2.16851161751063	2.60258466118318	0.34588158953537
H	2.29994832300626	3.67648465435787	0.49841697706419
H	3.07249849901251	2.11464446710357	0.72688777453903
O	2.03150329666181	2.41632358793645	-1.04417853281700
H	2.03496963277824	1.46230557049174	-1.23383761631119
Cl	-2.55211181155542	0.60717597635257	3.55512980794881

Tab. S14: xyz coordinates for *p*ClT p at B3LYP-D3(BJ)/def2-QZVP level.

atom	x	y	z
C	-2.63642171033486	-2.48506113296900	-1.35614073438914
C	-2.88491216836952	-1.04481194971217	-1.77856350878868
C	-1.70380299695817	-0.10602527607891	-1.49535251374291
C	-0.14471871520296	-2.21643348807596	-1.85762137716860
C	-1.42785530342478	-3.01868686550482	-2.11038659145334
H	-2.48301295277230	-2.55246880574234	-0.27814993486025
H	-3.51247505930367	-3.09450469628423	-1.58052636319664
H	-3.09735397996314	-1.02607128310677	-2.85036001813539
H	-3.76079853650268	-0.63235201772325	-1.27696009695984
H	-1.64713248872871	-2.99783922662102	-3.18059821575278
H	-1.22651053187654	-4.05861860667705	-1.85091903524917
C	-1.89177241145574	1.18768234829728	-2.29198868397894
H	-1.16500227375101	1.94593872413870	-2.01832987691939
H	-2.88659322845404	1.58186784987942	-2.08587465875817
H	-1.81285076967477	0.99639084141793	-3.36114893312608
C	-1.59856479749212	0.22534930334062	0.00050873118115
H	-1.58776426029891	-0.66467840723012	0.62414602177785
H	-2.45395861256647	0.83226913418923	0.29559113419838
H	-0.69469884982316	0.79320609017949	0.19618683975201
C	0.88560114080655	-2.55618133585208	-2.93650608316551
H	1.82864303099660	-2.05045626274838	-2.75332876223888
H	0.52287983548875	-2.26114202887789	-3.92008648402416
H	1.05596308346030	-3.63234958820345	-2.93891739039247
C	0.44556005164256	-2.52541172041056	-0.47326208823361
H	1.29013544191382	-1.86944234894243	-0.27936031672811
H	0.79203375115413	-3.55819535839565	-0.44829682100736
H	-0.27969376615407	-2.39196923788964	0.32509980742083
N	-0.42822064159901	-0.74841925978604	-1.94859031391116
O	0.60274751341898	0.00264404528540	-2.03060410395838
C	1.60259653545872	0.06966657779794	2.15468702720752
C	2.11434829495666	0.69270933407294	1.02315193000765
C	0.72315808445510	0.76811088866025	2.96658719069466
H	2.78867211308464	0.14818241517067	0.37635417033315
C	1.75942600664380	1.99713910471915	0.69448929917342
C	0.35931277500389	2.07553378407353	2.67040335577528
H	-0.32342558120022	2.60504824241487	3.31763721857149
C	0.88085500454982	2.67905032292886	1.53602092056535
H	0.58877116850352	3.69102609887586	1.29004357928958
H	1.87458669116448	-0.94564828486170	2.40052509676416
C	2.24873416877308	2.63772359492532	-0.58101915846628
H	3.12632436701000	2.09621538200818	-0.94927951512262
H	2.55072040904099	3.66822989544310	-0.39318763859996
O	1.23606827293052	2.69859235600298	-1.57325732387297
H	0.96401459990672	1.78867507906418	-1.78184032533947
Cl	0.05548717246108	-0.01028147575639	4.38145337599952

3.3 *o*ClT

The xyz files for the three most stable *o*ClT complexes (Fig. 8 in the main publication) are listed in Tab. S15, Tab. S16, Tab. S17 and Tab. S18. Additionally, the coordinates of *o*ClT o” are shown in Tab. S19.

Tab. S15: xyz coordinates for *o*ClT o at B3LYP-D3(BJ)/def2-QZVP level.

atom	x	y	z
C	-3.78453034612296	-0.51710379161557	-0.61423692425790
C	-2.56745783212031	-0.14718190976855	-1.44956497275725
C	-1.75851459679950	1.02330808073125	-0.87512216171146
C	-2.57365387719345	0.22766128684126	1.51153718619438
C	-3.32957901193732	-0.89460247111249	0.78818077593380
H	-4.30712727330109	-1.35749289544621	-1.07246558967423
H	-4.49909868239987	0.30682451992084	-0.58139758810575
H	-2.85346094581553	0.11440260217033	-2.46895612459649
H	-1.91074115192168	-1.01733042210066	-1.51826822577476
H	-4.17517661069944	-1.18388774014193	1.41330394793637
H	-2.67326967334023	-1.76544369411579	0.72029196093759
C	-2.47073672322411	2.36781862535803	-1.09266649495893
H	-3.50024529796869	2.35495136004390	-0.74466365638139
H	-1.93714583563507	3.15731115595187	-0.56756200652190
H	-2.47823215028998	2.60366226490729	-2.15628072185516
C	-0.38748783721557	1.06236279797453	-1.54969182633355
H	0.15455561587613	0.13787003681008	-1.37381647457668
H	-0.52710795680219	1.18036343125675	-2.62363581285688
H	0.20994270167346	1.89138185260829	-1.18280646467447
C	-3.52529879416166	1.34074328741980	1.97977488617259
H	-4.16892632170478	1.70089721543443	1.18181303650483
H	-4.16112527169622	0.95948679041336	2.77822616133578
H	-2.95008313686086	2.17928490102989	2.36657630802313
C	-1.85050949598297	-0.35262939005410	2.72732100439389
H	-1.35332257744426	0.42146813050569	3.30387651844135
H	-2.58020190069030	-0.84715363546660	3.36736647820051
H	-1.10814249341258	-1.08666151854611	2.42330717033686
N	-1.54441683663777	0.81614337972580	0.59463359742603
O	-0.65330053193690	1.55157245126375	1.14059105723500
C	0.95505952996954	-2.64249936282619	-0.38541916180880
C	1.27261312505435	-1.65865340418756	0.54080245959356
C	1.56834966335915	-2.64604366404567	-1.63198805264015
H	0.80457190802947	-1.64158527254146	1.51242384511219
C	2.19635146117692	-0.65765165085809	0.25235078676639
C	2.50712660302251	-1.67035622571665	-1.93930978328803
H	3.00059741795150	-1.65583809815239	-2.89944096872444
C	2.81073384576243	-0.69730521833066	-0.99785580339150
H	0.23155123330474	-3.40544145666967	-0.13394190663158
C	2.49149880643130	0.43973245273255	1.25427147515712
H	3.50438924428817	0.31074385110582	1.64165904531099
H	2.47592814760860	1.40182628243114	0.73517807219023
O	1.61834136282186	0.44589686823875	2.35924969159087
H	0.78041072747941	0.84421665375161	2.07111335277137
H	1.32586863662566	-3.40419158973212	-2.36301014479977
Cl	3.99396433535705	0.52308751851961	-1.42100322759441

Tab. S16: xyz coordinates for *o*ClT t at B3LYP-D3(BJ)/def2-QZVP level.

atom	x	y	z
C	-3.72549979123329	1.27445411496465	1.27727067411839
C	-4.14972634212388	0.01654285449795	0.53297344113520
C	-2.97100163766421	-0.82072550742812	0.01667947562090
C	-1.63601796438910	1.46075286112720	-0.18151729608016
C	-2.90228052422393	2.14991289675301	0.34313499815699
H	-3.15721228422215	1.02241244482112	2.17384984214199
H	-4.60742110156911	1.81934510224213	1.61572072752468
H	-4.76874287771433	0.30790084014502	-0.31896475986623
H	-4.76446746697100	-0.62692596804958	1.16312027400094
H	-3.52452386335965	2.43418004855996	-0.50897972883234
H	-2.59881776579481	3.07495445300888	0.83429750976706
C	-3.47447776515983	-1.80827788750797	-1.03807025416885
H	-3.87425432220832	-1.27847898996571	-1.90140003318020
H	-2.68069098367012	-2.46743593189871	-1.37601383997360
H	-4.26978869571172	-2.41362019458007	-0.60436894444458
C	-2.29548343650226	-1.59856755451809	1.15732832884701
H	-1.43131231462868	-2.14308438667033	0.78411141387411
H	-1.97555119006462	-0.94919351639633	1.96792713672459
H	-3.00083749863994	-2.32261448394853	1.56422895732783
C	-1.09256802325157	2.23036361991376	-1.38680687865015
H	-1.80560944666023	2.21100993283793	-2.20986273519622
H	-0.92594621550133	3.26734521731840	-1.09685322837240
H	-0.15373428521427	1.80768708339493	-1.73120638425420
C	-0.54766893754269	1.38850161694641	0.90068100205191
H	0.30501123904085	0.82484073406044	0.53413735664248
H	-0.20947481623322	2.39623184454197	1.13959649046953
H	-0.90265131605298	0.92763130292620	1.81863211115363
N	-1.96657481389243	0.07364441399113	-0.64061527994621
O	-1.09588108033499	-0.48956935028391	-1.38635303078647
C	2.92134405331909	0.78539562231836	-2.08342173645717
C	2.24797301512814	-0.35557268458066	-1.66922650052005
C	3.62735671021825	1.55019067700709	-1.16368379366224
H	1.69020199198384	-0.94880735374625	-2.37711205888714
C	2.25916225120316	-0.75995470674964	-0.33661178265401
C	3.66107758657970	1.16597588512510	0.16983702884736
H	4.20439740371193	1.74314915850773	0.90279679288968
C	2.98210199870952	0.02210098225129	0.56284309912135
H	2.89198000593677	1.07748323314095	-3.12360730057902
C	1.49250991361730	-1.98324740632471	0.12062320798616
H	2.19714227825270	-2.75210098851695	0.44285144047382
H	0.90731770989136	-1.71417195901931	1.00685165431526
O	0.67968432288078	-2.56339146745064	-0.87064869234857
H	-0.01933565035822	-1.92828344654774	-1.10222016392006
H	4.15219531388809	2.44149690046807	-1.47696601602912
Cl	3.03371482833428	-0.42985660972803	2.25669508195375

Tab. S17: xyz coordinates for *o*ClT o' at B3LYP-D3(BJ)/def2-QZVP level.

atom	x	y	z
C	-4.04741320306461	-0.50202525717334	0.43785011748061
C	-2.77719842536993	-1.16119440400692	0.95425028224389
C	-1.69866903624148	-0.16245202296259	1.39428260178625
C	-2.69052292115055	1.48104118317979	-0.43533409720775
C	-3.69785334908254	0.37006690022525	-0.75903732479272
H	-4.52690369412282	0.08696328964886	1.22120923939132
H	-4.76810152637440	-1.26534093535877	0.14274655994433
H	-2.36509897986104	-1.79182365145673	0.16316834611469
H	-2.98678694606784	-1.81639719940520	1.80036969152075
H	-3.27763222204760	-0.26455029524689	-1.54289202147656
H	-4.58716240795947	0.84110400011748	-1.17915130600077
C	-0.35434345498276	-0.88446044828505	1.49189148540840
H	-0.07332169694953	-1.30533330417091	0.52903529283129
H	0.43561501202535	-0.21514344743961	1.81682555191446
H	-0.44106315872399	-1.70117968800665	2.20759457823790
C	-2.04031264407908	0.47455913317164	2.75012092034293
H	-3.03783865003711	0.90732415867672	2.76358470184095
H	-1.99174227574064	-0.28536530748199	3.52924477272584
H	-1.32016474504237	1.25601734605680	2.98207310151193
C	-2.11136686806188	2.03392791247584	-1.73853320722372
H	-1.45159059879890	2.87676274585447	-1.55822310290072
H	-1.55023935164819	1.26716586031491	-2.27002003075918
H	-2.93010891941094	2.36462690609553	-2.37668500737627
C	-3.34922251803555	2.62265957580133	0.35501728194684
H	-2.59202411686742	3.33551392491850	0.67381458174603
H	-4.06577856009279	3.13879737400670	-0.28313541668051
H	-3.87875966410552	2.26377552008358	1.23412516716405
N	-1.55675692682673	0.92653726756198	0.37296619274575
O	-0.54454456971780	1.69333229364682	0.52172174785159
C	3.74710988709694	0.04484787599896	1.42114888635329
C	3.01579689885510	0.64794250188915	0.40693558569965
C	3.88800614978534	-1.33669414942344	1.45126883568337
H	2.89398955097296	1.72001194370984	0.37724923828977
C	2.41009051593047	-0.10488070935961	-0.59539231180604
C	3.29830433786703	-2.11060360592043	0.46101691298633
H	3.39310512667534	-3.18605331744576	0.46344866973150
C	2.57382963407962	-1.48714242663542	-0.54367710024723
H	4.20460966489290	0.65363586419647	2.18817618683560
C	1.58973621399586	0.55820781541570	-1.68187226286240
H	2.10092530108475	0.44351654216447	-2.63991471672298
H	0.63698115613719	0.02650740370458	-1.77475163135570
O	1.38303209033405	1.93539714629924	-1.48242974784059
H	0.70894468227652	2.02928832932009	-0.78927877325723
H	4.45310827893663	-1.81517704178444	2.23847924583807
Cl	1.82405171113202	-2.49448558708938	-1.77006123165610

Tab. S18: xyz coordinates for *o*ClT t' at B3LYP-D3(BJ)/def2-QZVP level.

atom	x	y	z
C	-3.49739925355884	1.99335587100171	0.92578215253227
C	-3.21541865101491	0.67191772643292	1.62653533747966
C	-1.95859279325424	-0.03816602127975	1.10700217607492
C	-2.46224308593252	1.09124154802283	-1.23403221162759
C	-3.68232108775322	1.73806569187698	-0.56318549327339
H	-2.68848561703000	2.70439319779546	1.10008267823081
H	-4.39932083756570	2.44736034893298	1.33778254802810
H	-4.07419631886114	0.01084933421503	1.48638378723794
H	-3.09794440777079	0.81285077573136	2.70147587168952
H	-4.54374342058522	1.07989167386023	-0.69990684783577
H	-3.90490890167432	2.66239183378521	-1.09741176106507
C	-1.96474237333682	-1.49592256146011	1.57035084980918
H	-2.81108742369265	-2.03274464982119	1.14407081026952
H	-1.05121038750294	-2.00505996416645	1.27891060662527
H	-2.04959356464707	-1.52092364476375	2.65627502810535
C	-0.67828708307915	0.64655458069083	1.60845173765497
H	-0.66972593978131	1.71358493522072	1.40334440417739
H	-0.59558926732531	0.51041965242177	2.68628420123646
H	0.19388853399910	0.20208559254263	1.13873689191911
C	-2.87929009468681	0.51394724125937	-2.58850031789847
H	-3.60459000288412	-0.28764940766822	-2.45779539972973
H	-3.33844291234915	1.30326371190705	-3.18280665776343
H	-2.02828742060678	0.12029720836210	-3.13611124642908
C	-1.33053766097025	2.10973792586297	-1.44091544208911
H	-0.44140851364101	1.61120309134053	-1.81813330268625
H	-1.64452715140639	2.85421539426208	-2.17206005329477
H	-1.06734308156567	2.62742749794383	-0.52237489931477
N	-1.96266535045851	-0.03956783093245	-0.39103840852578
O	-1.16094118332260	-0.86120748882866	-0.95008230674315
C	2.67562692011711	1.82984812394907	0.16929427629884
C	2.28460311466125	0.91177960053354	-0.79426913753804
C	3.10433802561814	1.38839900876868	1.41495051303937
H	1.94251352947536	1.24113235345531	-1.76361550196704
C	2.30784689485941	-0.45973482971885	-0.54774493343406
C	3.14783127152983	0.02798434066702	1.68470596342473
H	3.47881860986054	-0.33841855250255	2.64478483514957
C	2.75597000209045	-0.87448522042833	0.70508968490276
H	2.64427267941282	2.88768095186510	-0.05098216485198
C	1.85884145987608	-1.43874395028599	-1.61334855094099
H	2.73769466540336	-1.85697364073850	-2.10958299490828
H	1.33572020109914	-2.26979223777624	-1.13825935391323
O	1.05731281646614	-0.84568457032016	-2.61332638207397
H	0.16690378037336	-0.76263278744564	-2.23661752506486
H	3.40625755184578	2.09449504022183	2.17541630218531
Cl	2.82962843803210	-2.58361996404051	1.08734811136653

Tab. S19: xyz coordinates for *o*ClT o" at B3LYP-D3(BJ)/def2-QZVP level.

atom	x	y	z
C	-2.81140039966841	2.04901359358792	0.51482725308940
C	-2.08270839794762	1.62481875535353	-0.75234444445992
C	-2.02327752762821	0.10413625235704	-0.94612499737838
C	-2.04087422043493	-0.08880068415147	1.69032511720894
C	-2.10564058228866	1.44416139648541	1.72003095024708
H	-3.85831388096503	1.74309650528681	0.48204893606407
H	-2.81227917201806	3.13662051063379	0.59507929251089
H	-1.06064200248541	2.00829828289590	-0.71404028344618
H	-2.54939737907945	2.05536214421949	-1.63916399349525
H	-1.08541619915200	1.83154439468653	1.75744849753337
H	-2.59174938917370	1.73871021339244	2.65094877080915
C	-0.97786675506889	-0.22883307065284	-2.00959068145471
H	0.00637663885122	0.11138074761244	-1.70264817801566
H	-0.93091659075130	-1.29727029713495	-2.19585960443857
H	-1.24336956352329	0.27867083793795	-2.93636347764450
C	-3.38366554906556	-0.46681219024324	-1.37750688137821
H	-3.34407648147362	-1.55409357823125	-1.37719958454636
H	-4.19486570301407	-0.14924084624928	-0.72788354164593
H	-3.61105395967535	-0.12928870117287	-2.38821305943561
C	-1.00845327837913	-0.56768827174654	2.71080474467396
H	-0.93508989130238	-1.65099689727321	2.72208877123167
H	-0.02972524021409	-0.14987227776976	2.48757958498000
H	-1.30612710895717	-0.22543692987551	3.70126929471247
C	-3.40336395137770	-0.72182520327220	2.01581505715463
H	-3.35661871806434	-1.79809683347454	1.86350025340497
H	-3.65030064399599	-0.52990511429030	3.05946511898843
H	-4.20497823006311	-0.31956444316228	1.40234351252974
N	-1.59915312482651	-0.55159590597282	0.33405496203975
O	-1.23919754815839	-1.77439528520303	0.24445183294124
C	2.70117410741614	-0.54659708738363	-2.78346100776160
C	2.52509495962839	-1.29922495626583	-1.63381662024935
C	2.69975711106238	0.84200835061801	-2.70955942659738
H	2.49992841524539	-2.37820391878298	-1.68155545750136
C	2.34800496177327	-0.70055950473198	-0.38511356662294
C	2.52332194946959	1.46521679880641	-1.48405425235964
H	2.51435212175732	2.54176358939036	-1.40429831227581
C	2.35221570045980	0.69131633871932	-0.34181948810239
H	2.83414504969680	-1.03972238445549	-3.73586881388013
C	2.16643570439405	-1.57962714318296	0.83545496351054
H	3.14701389889101	-1.92103093403600	1.17453311365748
H	1.73508528817112	-0.99886080546105	1.64906485577538
O	1.40747547683590	-2.74036746081843	0.57305421126771
H	0.48453246549276	-2.47146338927514	0.43277929527848
H	2.83129411149719	1.44034161844530	-3.59983039306174
Cl	2.12735199391196	1.54270324324836	1.17825909981060

References

(S1) Neese, F. Software update: The ORCA program system—Version 5.0. *WIREs Comput. Mol. Sci.* **2022**, *12*, e1606.

(S2) Johnson, E. R.; Keinan, S.; Mori-Sánchez, P.; Contreras-García, J.; Cohen, A. J.; Yang, W. Revealing Noncovalent Interactions. *J. Am. Chem. Soc.* **2010**, *132*, 6498–6506.

(S3) Lu, T.; Chen, F. Multiwfn: A multifunctional wavefunction analyzer. *J. Comput. Chem.* **2011**, *33*, 580–592.

(S4) Humphrey, W.; Dalke, A.; Schulten, K. VMD: Visual molecular dynamics. *J. Mol. Graph.* **1996**, *14*, 33–38.

(S5) Lange, M.; Sennert, E.; Suhm, M. A. Attaching Onto or Inserting Into an Intramolecular Hydrogen Bond: Exploring and Controlling a Chirality-Dependent Dilemma for Alcohols. *Symmetry* **2022**, *14*, 357.
